# A Structure‐Preserving, Dimensionality‐Increasing Strategy for the Stepwise Synthesis of Microporous α‐MoO_3_ with a Broad (100) Surface

**DOI:** 10.1002/anie.202506758

**Published:** 2025-06-23

**Authors:** Takuo Minato, Misato Miyamoto, Satoshi Ishikawa, Norihito Hiyoshi, Makoto Maeda, Kenji Komaguchi, Masahiro Sadakane

**Affiliations:** ^1^ Department of Applied Chemistry Graduate School of Advanced Science and Engineering Hiroshima University 1‐4‐1 Kagamiyama Higashi‐Hiroshima Hiroshima 739‐8527 Japan; ^2^ Department of Material and Life Chemistry Faculty of Engineering Kanagawa University 3‐27‐1 Rokkakubashi, Kanagawa‐ku Yokohama Kanagawa 221‐8686 Japan; ^3^ Research Institute for Chemical Process Technology National Institute of Advanced Industrial Science and Technology 4‐2‐1 Nigatake, Miyagino‐ku Sendai Miyagi 983‐8551 Japan; ^4^ Natural Science Center for Basic Research and Development Hiroshima University 1‐3‐1 Kagamiyama Higashi‐Hiroshima Hiroshima 739‐8530 Japan; ^5^ Present address: Materials and Structures Laboratory, Institute of Integrated Research Institute of Science Tokyo 4259 Nagatsuta‐cho, Midori‐ku Yokohama Kanagawa 226‐8501 Japan

**Keywords:** Crystal engineering, Molybdenum oxides, Organic–inorganic hybrid composites, Polyoxometalates, Synthesis design

## Abstract

Metal oxides with diverse structures and dimensionalities are typically synthesized via solid‐state or hydrothermal reactions. However, it is quite difficult to retain the structures of the starting materials when 0D metal salts or molecular clusters are used as precursors because higher‐dimensional structures form by structural reorganization through isomerization and decomposition/condensation reactions. In this study, we demonstrated a structure‐preserving, dimensionality‐increasing strategy for the synthesis of 2D α‐MoO_3_ from 0D [Mo_2_O_5_(H_2_O)_6_]^2+^ species via a 1D [Mo_2_O_6_{(CH_3_)_2_NCHO}]*_n_* intermediate while maintaining the structures of the precursors. By simple temperature‐controlled calcination, metastable crystals of α‐MoO_3_ in which the (100) plane was the broad face were successfully synthesized, differing from conventional α‐MoO_3_ in which the (010) plane is the broad face. In addition, the prepared metastable crystals possessed large surface areas, unusual micropores, and surface‐exposed coordinatively unsaturated Mo sites, allowing them to serve as high‐performance acid catalysts. This synthesis strategy is expected to facilitate the synthesis of metastable structures and the design of defects and surface structures at the atomic level.

## Graphical abstract

A structure‐preserving, dimensionality‐increasing strategy was developed to synthesize 2D α‐MoO_3_ from 0D [Mo_2_O_5_(H_2_O)_6_]^2+^ via a 1D Mo_2_O_6_{(CH_3_)_2_NCHO} intermediate. Unlike conventional α‐MoO_3_, which predominantly exposes the (010) plane, simple temperature‐controlled calcination of the 1D structure yielded metastable α‐MoO_3_ with the (100) plane as the broad face, large surface areas, and micropores, enabling high‐performance acid catalysis.

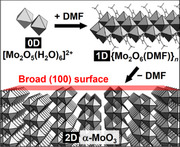

## Introduction

**Figure 1:**
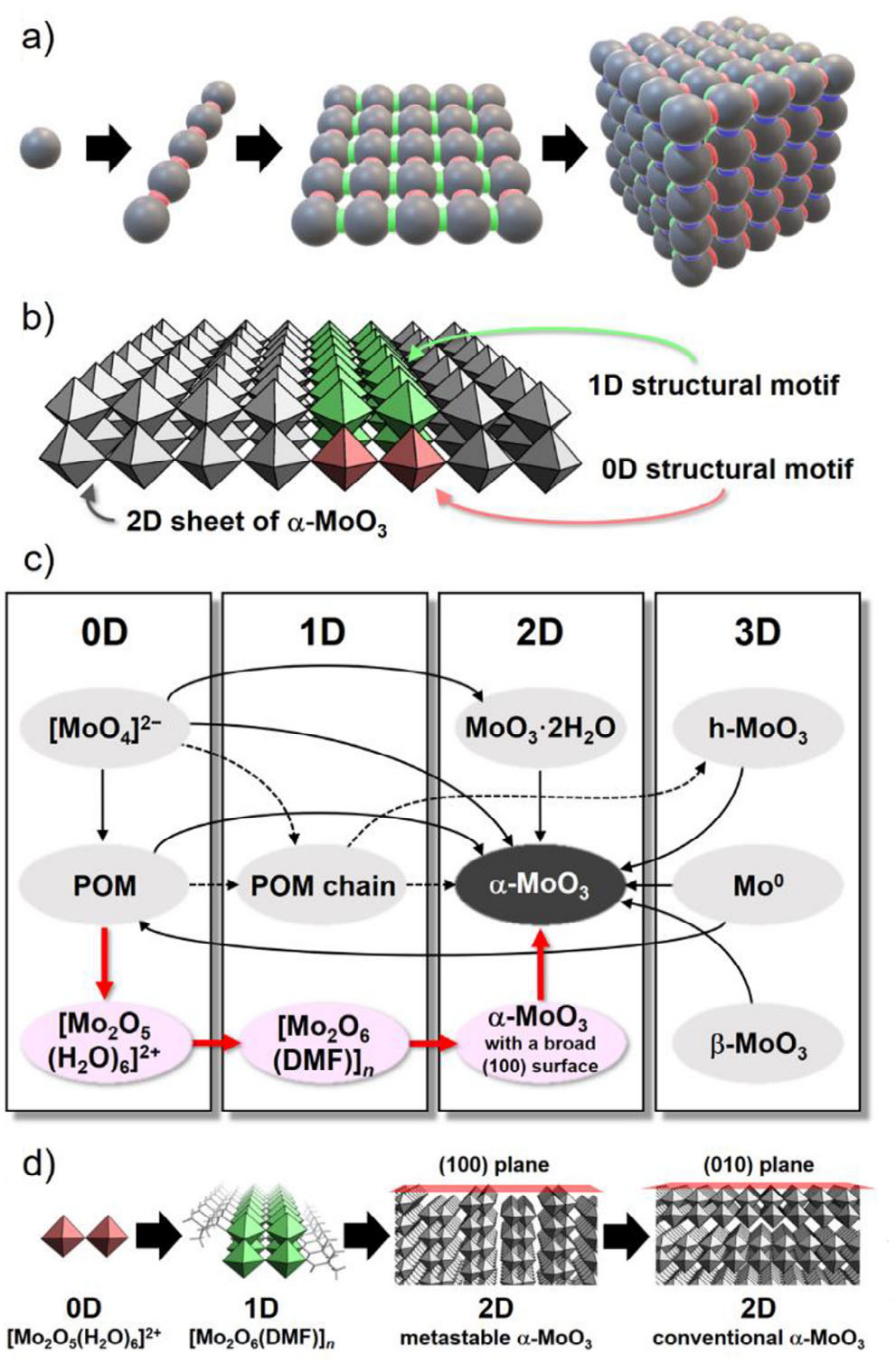
a) Schematic diagram of the structure‐preserving stepwise dimensionality‐increasing strategy for synthesizing a 3D compound from 0D precursors via 1D and 2D intermediates with retention of the precursor structures. The gray balls represent 0D atoms or molecules as a minimum structural motif of the desired 2D or 3D material. b) Polyhedral representation of the 0D and 1D structural motifs of 2D α‐MoO_3_ used in this study. c) Conventional synthesis route to α‐MoO_3_ (black arrows), a recently investigated dimensionality‐increasing synthesis route (dashed black arrows), and the structure‐preserving dimensionality‐increasing synthesis route developed in this study (red arrows). d) Structural evolution demonstrated in this study.

The spatial dimensionality (0D, 1D, 2D, or 3D) of a compound is an important parameter for predicting and determining the chemical and physical properties of the compound.^[^ ^1^, ^2^, ^3^, ^4^, ^5^, ^6^ ^]^ By systematically and gradually increasing the structural complexity and dimensionality of metal oxides, metastable 2D and 3D structures can be synthesized, and defects and surface structures can be designed at the atomic level, allowing control of their conductivity, porosity, catalytic activity, and magnetic properties (Figure 1a). However, this strategy remains quite challenging because most 2D and 3D bulk oxides are constructed from 0D metal salts or molecular clusters via solid‐state or hydrothermal reactions without the formation of 1D or 2D intermediate structures.^[^ ^7^, ^8^, ^9^, ^10^, ^11^ ^]^

Molybdenum oxides exhibit diverse structures and dimensionalities, including α‐MoO_3_ (2D); β‐MoO_3_ (3D); h‐MoO_3_ (3D); reduced phases such as Mo_8_O_23_ (3D), Mo_18_O_52_ (quasi‐2D, derived from α‐MoO_3_ via crystallographic shearing), and MoO_2_ (3D); and anionic molecular metal oxide clusters (polyoxometalates, POMs) such as [Mo_7_O_24_]^6−^ (0D) and [Mo_36_O_112_(H_2_O)_16_]^8−^ (0D).^[^ ^12^, ^13^, ^14^ ^]^ Among these structures, α‐MoO_3_ possesses a unique 2D sheet structure consisting of edge‐sharing octahedral {MoO_6_} zigzag chains connected along the [100] direction by corner‐sharing Mo─O─Mo bonds (Figure 1b). The 2D sheets in α‐MoO_3_ are held together by van der Waals interactions along the [010] direction, so α‐MoO_3_ can be used as an electrode material through intercalation.^[^ ^15^, ^16^ ^]^ Additionally, because of its highly electrophilic lattice oxygen atoms, α‐MoO_3_ serves as an active and selective catalyst for the oxidation of alcohols and hydrocarbons.^[^ ^17^ ^]^ In conventional plate‐like crystals of α‐MoO_3_, the (010) plane is typically the broad face; however, the high oxygen vacancy formation energy makes the oxygen vacancy at the (010) plane unstable or difficult to form (Figure S1).^[^ ^18^ ^]^ In contrast, the (100) plane of α‐MoO_3_ has a lower oxygen vacancy formation energy and exhibits unique catalysis and adsorption properties.^[^ ^19^, ^20^, ^21^, ^22^, ^23^ ^]^ Additionally, the (100) plane is the layer‐exposed surface, which is beneficial for guest intercalation. Therefore, the exposed surfaces should be controlled to achieve high‐performance electrode materials and catalysts. Nevertheless, the synthesis of α‐MoO_3_ in which the (100) plane is the broad face has been quite difficult because of the intrinsic structural anisotropy of this material and because of variation in the crystal growth rate (*r*_hkl_) along the principal directions: *r*_001_ > *r*_100_ > *r*_010_.^[^ ^24^, ^25^ ^]^

The challenge in achieving precise oxide design stems from the difficulty of synthesizing low‐dimensional structural motifs of the desired 2D or 3D metal oxides and controlling the condensation reactions of the precursors. Various methods for the synthesis of α‐MoO_3_ typically involve 0D precursors, such as monomolybdate [MoO_4_]^2−^ or POMs, resulting in the conversion of 0D structures to 2D or 3D structures (Figure 1c). To determine the intermediate structures, the temperature‐controlled calcination of 0D [MoO_4_]^2−^ or [Mo_7_O_24_]^6−^ has been studied,^[^ ^26^, ^27^, ^28^, ^29^ ^]^ and the results showed that 1) dehydrative condensation of [MoO_4_]^2−^ formed discrete 0D [Mo_7_O_24_]^6−^ and [Mo_10_O_34_]^8−^ or polymeric 1D {[Mo_2_O_7_]^2−^}*_n_* and {[Mo_3_O_10_]^2−^}*_n_*, 2) 3D h‐MoO_3_ was formed via 1D {[Mo_8_O_26_]^4−^}*_n_* depending on the types of counter cations, and 3) 3D h‐MoO_3_ or 1D {[Mo_8_O_26_]^4−^}*_n_* was transformed into thermodynamically stable 2D α‐MoO_3_. These findings demonstrated that the dimensionality increased as follows: 0D [Mo_7_O_24_]^6−^ → 0D [Mo_10_O_34_]^8−^ → 1D {[Mo_8_O_26_]^4−^}*_n_* → 2D α‐MoO_3_ (Figure 1c). However, structures of precursors cannot be retained through this process because higher‐dimensional structures were formed by structural reorganization through isomerization and decomposition/condensation reactions, and thus, precise defect design and surface structure control via a simple calcination process remained difficult.

To address the abovementioned issues, we proposed a structure‐preserving, dimensionality‐increasing strategy to form the metastable state of 2D α‐MoO_3_ by assembling simple 0D units into 1D structures, which were then formed into 2D sheets while maintaining the precursor structures (Figure 1c). In this study, we successfully synthesized fibrous 1D crystals of [Mo_2_O_6_{(CH_3_)_2_NCHO}]*_n_* (**1**), which possessed essentially the same building unit of a 2D α‐MoO_3_ sheet, from a structurally controlled 0D dinuclear Mo species as a minimum structural motif of α‐MoO_3_ (Figure 1b). By calcining **1** at low temperatures, α‐MoO_3_ in which the (100) plane was the broad face was successfully synthesized, resulting in the first microporous α‐MoO_3_ with unique catalytic properties (Figure 1d).

## Results and Discussion

### Synthesis of 1D Mo_2_O_6_{(CH_3_)_2_NCHO} from 0D Dinuclear Mo Species

**Figure 2:**
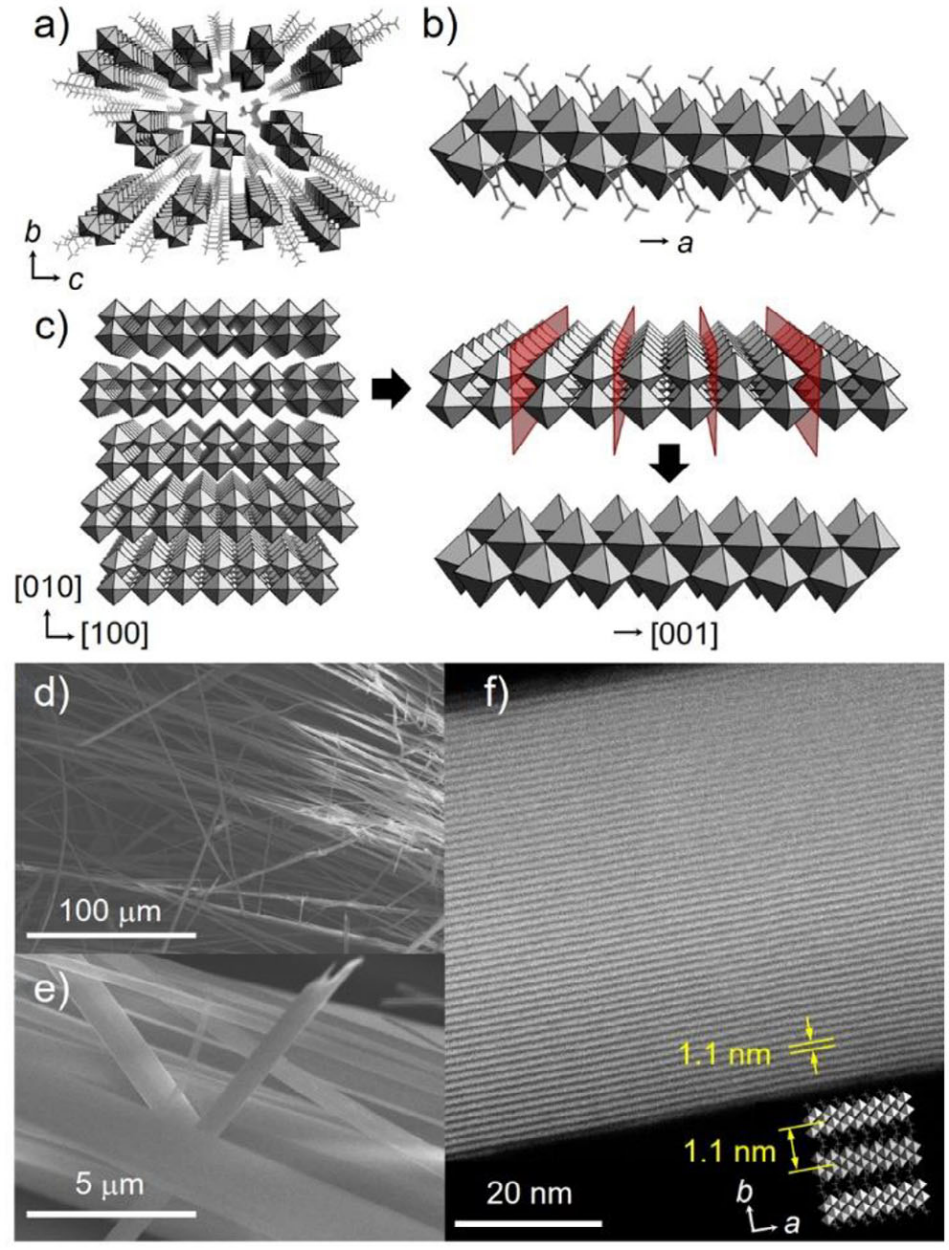
a) Crystal packing of **1** along the *a*‐axis. b) Crystal structure of subnanofiber **1**. c) Crystal structure of α‐MoO_3_. A 1D structural motif obtained by cutting the 2D sheet structure of α‐MoO_3_ along the red planes. d) and e) SEM images of microfiber **1**. f) STEM image of **1** projected from the *c*‐axis.

We focused on the dinuclear corner‐sharing [Mo_2_O_5_(H_2_O)_6_]^2+^ species ({Mo_2_}, Figures 1b and S2) as a minimum structural motif of α‐MoO_3_.^[^ ^30^, ^31^, ^32^ ^]^ Given that the edge‐sharing condensation reactions along the [001] direction of α‐MoO_3_ proceeded preferentially because of the high *r*_001_, the coordination of small molecules at both ends of {Mo_2_} suppressed the condensation reactions along the [100] direction and allowed the formation of 1D fibrous structures. Because monomolybdate [MoO_4_]^2−^ was converted into {Mo_2_} in a concentrated acidic solution, the addition of capping organic molecules into an acidic solution of [MoO_4_]^2−^ was examined. By vapor diffusion of *N*,*N*‐dimethylformamide (DMF) into a nitric acid solution of Na_2_MoO_4_, fibrous single crystals (**1**) were successfully obtained.^[^ ^33^ ^]^ X‐ray crystallographic analysis revealed that **1** belonged to the primitive monoclinic *P*2_1_/*c* space group, had a short *a*‐axis unit cell length, and existed as a bundle of unique 1D subnanofibers with a cross‐sectional area of 0.8 × 0.7 nm (Table S1; Figures 2a,b and S3). Each subnanofiber was composed of edge‐sharing zigzag chains of {Mo_2_}, which was essentially the same building unit obtained by cutting one layer of a 2D α‐MoO_3_ sheet into 0.8‐nm‐wide strips along the [001] direction (Figure 2c). Consequently, the dimensionality increased stepwise from 0D {Mo_2_} to 1D **1** while maintaining the structure of the precursor {Mo_2_}. Importantly, the O atoms in the DMF molecules were coordinated to the Mo atoms to prevent the subnanofibers from further condensation into a 2D structure. The infrared (IR) spectrum of **1** showed a sharp peak at 1664 cm^−1^ that was attributed to the stretching vibration of the C═O bond in DMF, which was shifted toward lower wavenumbers relative to that in the spectrum of free DMF (1677 cm^−1^), verifying the coordination of DMF molecules (Figure S4a).^[^ ^34^ ^]^ The bond valence sum values of Mo (5.92, 6.01) and O (1.75–2.12) indicated that the valence of Mo was 6+ and that the O atoms existed as oxo ligands (Table S2). On the basis of these results and the results of elemental analysis, the formula of **1** was determined to be Mo_2_O_6_{(CH_3_)_2_NCHO}. Crystals of **1** could not be obtained when using formamide, *N*‐methylformamide, or isobutyraldehyde as capping organic molecules (Figure S5). In the crystal packing of **1**, weak hydrogen bond networks CH_3_⋯O═Mo were observed (Table S3), indicating that two methyl groups of DMF were necessary to form **1**. In addition, DMF molecules were aligned along the *a*‐axis, indicating that the planar geometry of N was also important to form densely packed crystal structure **1**.

The scanning electron microscopy (SEM) images showed that **1** possessed a fibrous crystal morphology (Figure 2d,e). The scanning transmission electron microscopy (STEM) images of **1** displayed clear lattice fringes throughout the fibers with a lattice spacing of 1.1 nm, equal to half of the *b*‐axis unit cell length (1.1 nm) (Figure 2f). The STEM image of **1** projected from the [011] direction showed lattice spacings of 0.95 and 0.34 nm, indicating that bundles of subnanofibers grew along the *a*‐axis to form fibrous single crystals (Figure S6). The powder X‐ray diffraction (XRD) pattern of **1** agreed well with the simulated pattern of **1** by assuming a needle‐type crystal orientation along the *a*‐axis, supporting the high purity of **1** (Figure S7). In addition, the yield of **1** reached 95% based on Mo, even by gram‐scale synthesis, suggesting that the precursor {Mo_2_} was efficiently polymerized into **1**. The Raman spectrum of the synthesis solution of **1** at pH 0.8 exhibited bands centered at 903, 955, and 983 cm^−1^ that were attributed to [Mo_36_O_112_(H_2_O)_16_]^8−^ species (Figure S8).^[^ ^35^ ^]^ [Mo_36_O_112_(H_2_O)_16_]^8−^ was reported to be unstable in a highly concentrated acidic solution and to decompose into {Mo_2_} below the isoelectric point of molybdic acid at pH 0.9.^[^ ^30^, ^32^, ^36^, ^37^ ^]^ Therefore, single crystals of **1** were obtained presumably by 1) the formation of dinuclear corner‐sharing {Mo_2_} from [Mo_36_O_112_(H_2_O)_16_]^8−^, 2) partial replacement of aqua ligands by DMF molecules, and 3) the simultaneous condensation of {Mo_2_} and crystal growth of **1** along the *a*‐axis. These results indicated that unique 1D subnanofibers possessing essentially the same building unit of a 2D α‐MoO_3_ sheet with removable DMF molecules were successfully synthesized from {Mo_2_}. To date, edge‐sharing zigzag chains as 1D structural motifs of α‐MoO_3_, including {[Mo_3_O_9_(SO_4_)]^2−^}*_n_* and [Mo_2_O_7_(C_7_H_11_N_5_)]*_n_*, have been reported; however, **1** was the first example of a 1D structural motif of α‐MoO_3_ with a repeating {Mo_2_} unit (Figure 1b).^[^ ^29^, ^38^, ^39^, ^40^ ^]^

### Synthesis of 2D α‐MoO_3_ from 1D Mo_2_O_6_(DMF)

**Figure 3:**
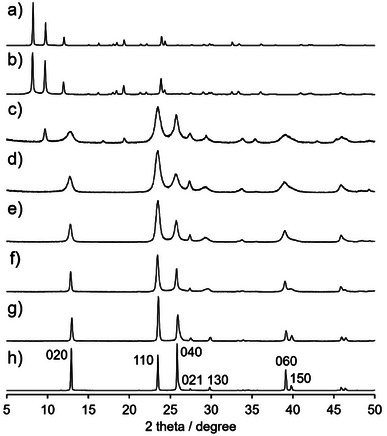
XRD patterns of a) **1**, b) **1_200_**, c) **1_250_**, d) **1_300_**, e) **1_350_**, f) **1_400_**, g) **1_500_**, and h) **1_600_**.

During calcination of anionic 1D POM chains, protons are necessary for dehydrative condensation to form 2D α‐MoO_3_, which induces reorganization of the precursors. In contrast, in this study, as the atomic arrangement of α‐MoO_3_ along the [001] direction was preformed in the neutral 1D structure of **1**, it was expected that the removal of DMF simply induced the condensation and stacking of subnanofibers, even in the absence of protons, without any reorganization of **1**. Therefore, a further stepwise increase in the dimensionality from 1D **1** to 2D α‐MoO_3_ was examined by heating the crystals of **1**. Thermogravimetric differential thermal analysis of **1** showed that weight loss occurred at 200–300 °C, and the 20.6 wt% weight loss at 600 °C was attributed to the loss of one coordinated DMF molecule per Mo_2_O_6_(DMF) (20.2 wt%) (Figure S9). Temperature‐programmed desorption (TPD) mass data showed that DMF and fragmented ion signals were observed at approximately 300 °C, verifying the removal of DMF from **1** (Figure S9). To confirm the dimensionality‐increasing process, **1** was heated at various temperatures (*n*°C) for 6 h in air to prepare calcinated samples **1*_n_***. The XRD pattern of **1_600_** showed sharp diffraction peaks that were attributed to α‐MoO_3_, indicating that structural transformation occurred via the removal of DMF molecules (Figure 3). Broad diffraction peaks attributed to α‐MoO_3_ were observed at 220 °C following the removal of DMF, and the structural transformation was completed at 300 °C (Figures 3 and S10). Elemental analysis of the calcinated samples also verified that the removal of DMF was completed at 300 °C (Figure S11). The weak diffraction peaks (2*θ* = 9.6°, 16.7°, and 19.3°) observed in the patterns of **1_225_–1_250_** might be attributed to the 100, 110, and 200 reflections of h‐MoO_3_, respectively. The small amount of the hexagonal phase was presumably formed by the long calcination time at low temperature and was completely transformed into α‐phase at 300 °C (Figure S10). Calcination of **1** at 300 °C for 10 min (**1_300_** **^10^**) resulted in rapid transformation into α‐MoO_3_ (Figure S12), indicating that the main transformation route to form 2D α‐MoO_3_ was the direct condensation and stacking of the subnanofibers.

**Figure 4:**
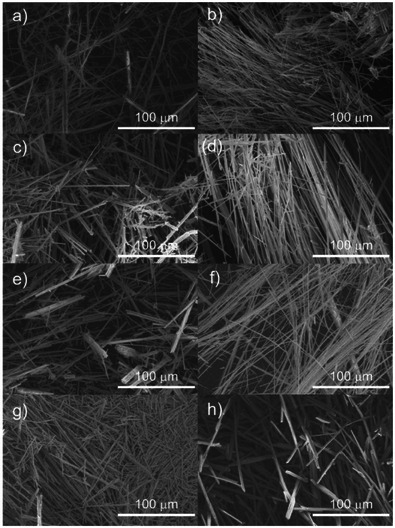
SEM images of a) **1_150_**, b) **1_200_**, c) **1_250_**, d) **1_300_**, e) **1_350_**, f) **1_400_**, g) **1_500_**, and h) **1_600_**.

The optical microscopy and SEM images of **1_150_–1_600_** clearly showed that the fibrous crystal morphology of **1** was retained during the calcination process, despite the removal of DMF molecules and the increase in the dimensionality of **1** (Figures 4 and S13). Notably, the crystal structure of α‐MoO_3_ contained 2D sheets, whereas **1_300_–1_600_** possessed a fibrous crystal morphology. Upon heating, the samples changed from colorless (**1**) to blue (**1_200_**) and then to off‐white (**1_600_**) (Figure S14a). The diffuse reflectance spectrum of **1_250_** showed absorption bands at 600 and 940 nm that were attributed to the intervalence charge transfer of Mo^6+^–O–Mo^5+^ (Figure S14b), suggesting partial reduction of Mo^6+^ to Mo^5+^. The intensities of these peaks decreased as the calcination temperature increased, indicating reoxidation of the Mo^5+^ species. Interestingly, **1_300_** contained Mo^5+^ species, although no diffraction peaks that could be attributed to reduced phases were observed (Figure 3). The electron spin resonance (ESR) spectrum of **1_300_** showed signals attributed to the F center (*g* = 2.0029) and the Mo^5+^ center (*g*_1_ = 1.9626, *g*_2_ = 1.9498, *g*_3_ = 1.8764) (Figure S15),^[^ ^41^, ^42^, ^43^ ^]^ and the numbers of electron spins in the samples were calculated from the double‐integrated spectra using the Mn^2+^/MgO signals measured together as a standard sample (Figure S16). Although signals could not be observed in the spectra of **1_500_** and **1_600_**, the stoichiometric deviations (*δ*) of MoO_(3−_ *_δ_*_)_ in **1_300_**, **1_350_**, and **1_400_** were estimated to be 4.8 × 10^−5^, 2.2 × 10^−5^, and 1.0 × 10^−5^, respectively. The Tauc plots generated from the diffuse reflectance spectra showed that the band gap energy of **1_300_** was small and increased as the calcination temperature increased, indicating the presence of oxygen defects in **1_300_–1_400_** (Figure S14c). A linear correlation was observed in a plot of the Kubelka–Munk value at 940 nm versus *δ*, indicating that the partial reduction of Mo^6+^ in α‐MoO_3_ was induced by the removal of lattice oxygen atoms (Figure S14d). On the basis of these results, **1_300_–1_400_** were characterized as the pure α‐phase of MoO_3_ with a small number of oxygen defects.

### Hierarchical Structure of 1_300_

**Figure 5:**
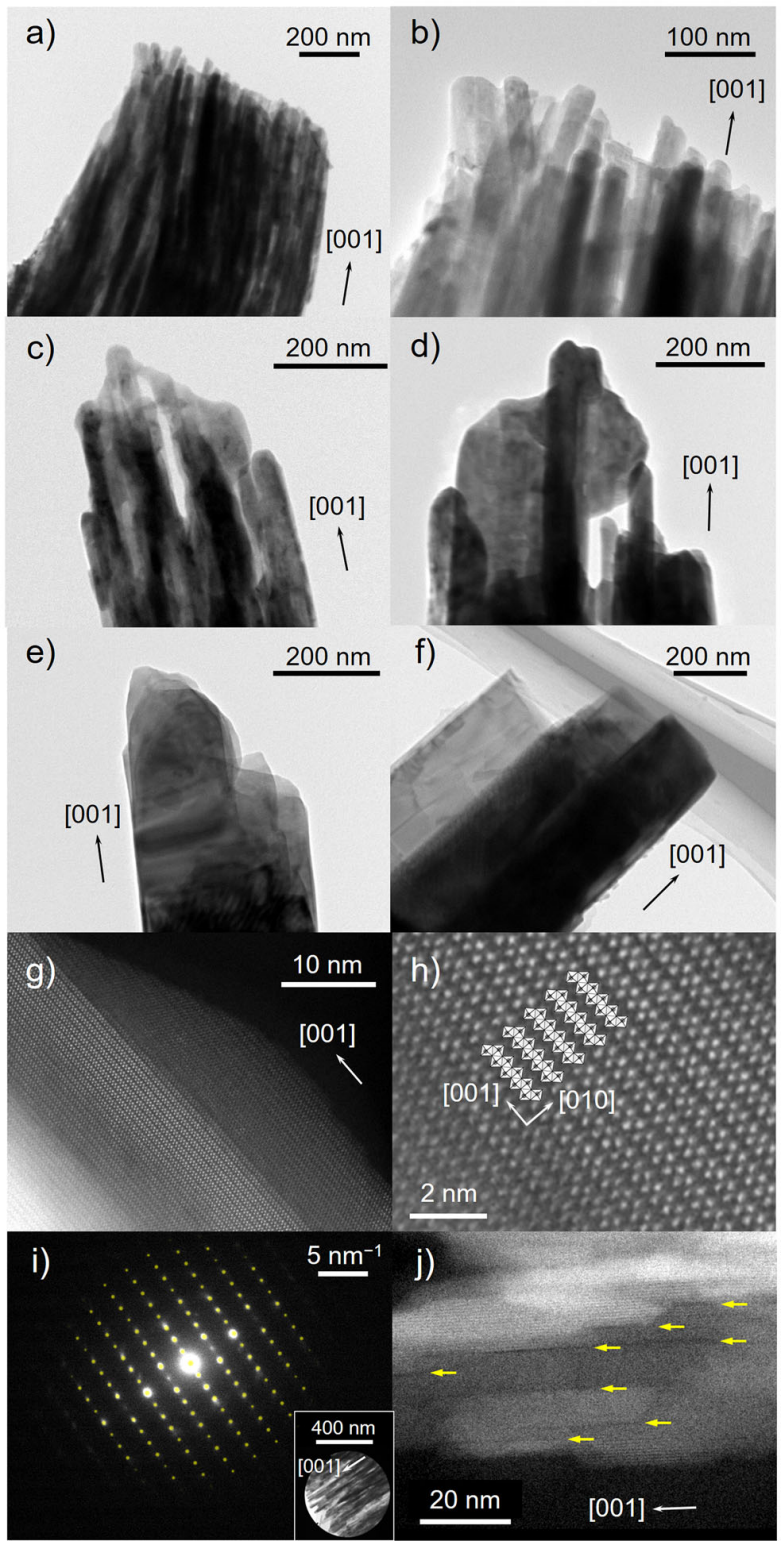
TEM images of submicroribbons a) and b) **1_300_**, c) **1_350_**, d) **1_400_**, e) **1_500_**, and f) **1_600_**. g) and h) STEM images of nanorods **1_300_**. i) TEM image and corresponding SAED pattern of submicroribbon **1_300_**. The superimposed yellow pattern represents the simulated diffraction pattern projected from the [100] direction. j) STEM image of submicroribbon **1_300_**. The yellow arrows indicate slit‐shaped micropores.

The peaks in the XRD patterns of **1_300_–1_600_** were attributed to α‐MoO_3_; the peaks were quite broadened for samples calcined at lower temperatures, and the diffraction peak attributed to the 110 reflection was unusually strong (Figures 3 and S17). To explain these observed phenomena, various types of microscopy were conducted. First, microfiber **1_300_** was dispersed in ethanol and sonicated to prepare submicroribbons, which were observed by transmission electron microscopy (TEM). The TEM image showed that each submicroribbon consisted of a bundle of finer nanorods, with an average width of 27 nm (Figures 5a,b and S18a). The lengths of the nanorods estimated from the TEM observations varied from 100 nm to over 1 µm. The nanorods were joined together longitudinally, and the resulting elongated nanorods then assembled laterally to form a bundle (Figure S19a,b). Next, to observe the cross‐section of **1_300_**, a microfiber **1_300_** was cut using a focused ion beam (FIB) SEM system. The FIB–SEM image showed cross‐sections of the randomly oriented submicroribbons, resulting in a porous structure (Figure S20a). All the submicroribbons within the fiber were fused to form a monolithic surface, presumably because the cross‐section was partly melted as a result of the high energy of the Ga ion beam. Nevertheless, plate‐ and lump‐shaped domains attributable to the large submicroribbons and possible crystallites of α‐MoO_3_, respectively, were observed (Figure S20b). On the basis of the FIB–SEM image, the porosity of the microfiber was calculated to be 0.41, which was similar to the calculated value of *V*(α‐MoO_3_)/*V*(**1**) = 0.48, where *V*(compound) is the unit cell volume of the compound per Mo, indicating that the DMF molecules were removed while maintaining the fibrous crystal morphology (Figure S21).

The STEM image of **1** showed that the tip of the fiber was not divided into finer rods, whereas the TEM image of **1_300_** **^10^** showed that the bundle of nanorods had formed within 10 min of calcination (Figure S22a,b). A bundle of nanorods also formed in **1_220_** as a result of DMF removal‐induced segmentation (Figure S19d,e). Interestingly, the average width of the **1_300_** **^10^** nanorods was 12 nm, whereas that of **1_300_** nanorods was 27 nm (Figure S18a), indicating that lateral fusion proceeded by increasing calcination time. The TEM image of **1_350_** showed that the average width of the nanorods increased to 51 nm (Figures 5c and S18a), indicating that further lateral fusion occurred when the calcination temperature was increased. The sizes of the crystallites of **1_300_** ^**10**^, **1_300_**, and **1_350_** were calculated by means of Scherrer's equation using the full width at half maximum (FWHM) of the 020 reflections (10.2, 15.3, and 26.0 nm, respectively), and these values were close to the average width of the nanorods (Figure S18b). The TEM images of **1_400_**, **1_500_**, and **1_600_** showed flat submicroribbons with widths of 200 nm to 1 µm that had formed via lateral fusion (Figure 5d–f). As the fusion reaction proceeded, the FWHMs of the diffraction peaks in the XRD patterns decreased, indicating that the broadening of the diffraction peaks of **1*_n_*** resulted from the formation of nanorods via the calcination of **1**.

Each nanorod in **1_300_** appeared to be a conventional plate‐like crystal in which the (010) plane was the broad face; however, the TEM image displayed clear lattice fringes with a lattice spacing of 0.7 nm, which corresponded to the interlayer distance of α‐MoO_3_ (Figure S19c). The STEM image of a nanorod in **1_300_** showed an ordered arrangement of bright spots, consistent with the ideal atomic arrangements of heavy Mo atoms projected from the [100] direction of α‐MoO_3_ (Figures 5g,h and S23). Interestingly, in the TEM image of a submicroribbon, the selected area electron diffraction (SAED) pattern showed diffraction spots that were attributed to the pattern projected from the [100] direction even though the submicroribbon consisted of a bundle of nanorods, indicating that these nanorods were oriented along not only the [001] direction but also the [010] direction to form submicroribbons in which the (100) plane was the broad face (Figure 5i). The 00*l* diffraction spots (*l* = odd), which represented systematic absences, were presumably attributed to the slightly curved nature of the actual surfaces with atomic‐height steps, as observed in the STEM images (Figure S23).^[^ ^19^, ^44^ ^]^ These results revealed the unusual broad (100) surface of the nanorods and the unique hierarchical structure of **1_300_** (microfibers ⊃ submicroribbons ⊃ nanorods) (Figure S24).

The random orientation of submicroribbons and isolated elongated nanorods within a microfiber in **1_300_** observed by FIB–SEM was induced by the presence of two orientations of subnanofibers **1** at right angles to each other along the *a*‐axis in the crystal packing (Figure 2a). The FIB–SEM image of **1_300_** also showed that small submicroribbons intersected orthogonally in a grid‐like pattern, verifying that the assembly of subnanofibers **1** proceeded randomly from the original crystal orientation (Figure S20c). These random orientations of submicroribbons with a broad (100) surface resulted in the highest intensity of the diffraction peak attributed to the 110 reflection in the XRD pattern of **1_300_** (Figure 3), while in the patterns of conventional α‐MoO_3_, this peak was not the most intense, mainly because *r*_100_ > *r*_010_ (Figure S25).

The broad face of nanorods **1*_n_*** was further analyzed by TEM. The SAED patterns of **1_220_**, **1_300_^10^**, **1_300_**, and **1_350_** showed that the observed surfaces were (100) planes, whereas in the SAED pattern of **1_400_**, the diffraction spots were attributed to the pattern projected from both the [100] and [010] directions (Figures S22c and S26). When the calcination temperature was increased, the SAED patterns of **1_500_** and **1_600_** showed diffraction spots that were attributed to the pattern projected from the [010] direction, where forbidden (100) and (001) reflections were observed, presumably because of the atomic steps on the surface observed by TEM (Figure 5f).^[^ ^45^ ^]^ These results showed that the broad face changed from the (100) plane to the (010) plane at approximately 400 °C, indicating that the stacking of the nanorods along the [010] direction and the condensation of the nanorods along the [100] direction were the dominant reactions below and above 350 °C, respectively. Sautet et al. determined the formation energies of bulk α‐MoO_3_ by using the double ribbon model (DMF‐removed subnanofiber **1**) via ab initio calculations, and the results suggested that condensation along the [100] direction resulted in a stabilization energy of 17.3 kcal mol^−1^.^[^ ^21^ ^]^ In contrast, stacking 2D α‐MoO_3_ layers along the [010] direction resulted in stabilization energies of 8–12 kcal mol^−1^. These findings are consistent with relative crystal growth rates in the order of *r*_001_ > *r*_100_ > *r*_010_ and surface energies in the order of (001) > (100) > (010),^[^ ^18^, ^24^ ^]^ while subnanofibers in this study were preferentially stacked along the [010] direction during calcination of **1** at temperatures below 350 °C. This phenomenon was attributed to the fact that the activation energy for the stacking reaction along the [010] direction via van der Waals forces was lower than that for the condensation reaction along the [100] direction via forming ionic bonds. Therefore, kinetically controlled metastable α‐MoO_3_ in which the (100) plane was the broad face could be formed by assembling confined subnanofibers within a microfiber at low temperatures. The SEM images of **1*_n_*** showed deterioration of the microfiber surfaces (**1_350_** and **1_400_**) and reconstruction of the crystalline surfaces with steps (**1_500_** and **1_600_**), indicating that condensation along the [100] direction affected the surface structure of the microfibers by decreasing the pores via reorganization and densification of the α‐MoO_3_ crystals (Figure S27). To date, various nanowires, nanoribbons, nanobelts, and nanorods of α‐MoO_3_ have been synthesized; however, the synthesis of ultrafine nanostructures with diameters <30 nm has been quite difficult.^[^ ^46^, ^47^, ^48^, ^49^, ^50^ ^]^ In this study, careful control of the calcination temperature and time resulted in the formation of ultrafine nanorods (Figure S18). Importantly, the broad face of these ultrafine nanorods was successfully tailored to expose the (100) plane even by gram‐scale synthesis although the synthesis of α‐MoO_3_ in which the (100) plane as the broad face has been quite difficult (Figure S24).

To investigate the formation mechanism of ultrafine nanorods, calcination of **1** at 300 °C for 6 (**1_300_^6^**) and 8 min (**1_300_^8^**) was performed. The XRD pattern of **1_300_^6^** showed diffraction peaks that were attributed to **1** although the 13.4 wt% weight loss was observed, whereas that of **1_300_^8^** showed diffraction peaks that were attributed to α‐MoO_3_ (Figure S12). Interestingly, the TEM image of **1_300_^6^** showed that the bundle of nanorods had formed before the structural transformation into α‐MoO_3_, indicating that the removal of DMF molecules was the key to form ultrafine nanorods (Figure S28). The ESR spectra of **1_300_^6^**, **1_300_^8^**, and **1_300_^10^** showed that the intensity of the signals assignable to four‐ and six‐coordinate Mo^5+^ centers decreased and increased, respectively, with increasing calcination time (Figure S29).^[^ ^51^ ^]^ Importantly, the intensity of the signal assignable to six‐coordinate Mo^5+^ centers in **1_300_^8^** was very weak even though the dominant phase of **1_300_^8^** was α‐MoO_3_, indicating that the stacking of DMF‐removed subnanofiber **1** along the [010] direction occurred at the initial stage of structural transformation into α‐MoO_3_. These results suggested the following formation mechanism of ultrafine nanorods with the broad (100) surface: 1) coordinatively unsaturated Mo sites were formed by removing DMF molecules from **1**, 2) ultrafine nanorods were formed because DMF molecules were removed while maintaining the fibrous crystal morphology (**1_300_^6^**), 3) α‐MoO_3_ was formed by stacking DMF‐removed **1** along the [010] direction (**1_300_^8^**), and 4) condensation reaction along the [100] direction proceeded (**1_300_^10^**).

### Microporous Surface Structures and Catalytic Properties of 1_300_

**Figure 6:**
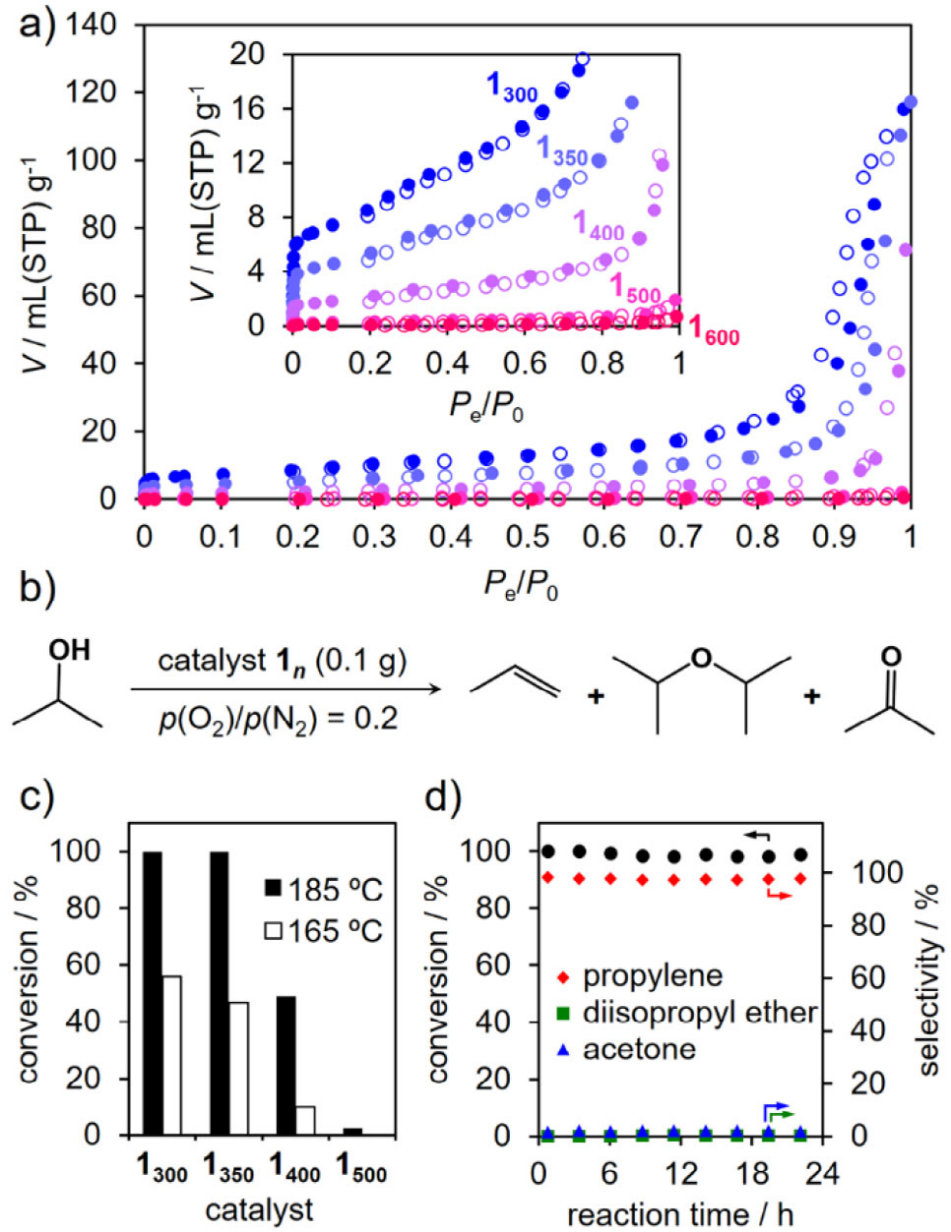
a) N_2_ sorption isotherms of **1_300_**, **1_350_**, **1_400_**, **1_500_**, and **1_600_**. b) Catalytic dehydration of 2‐propanol. c) Reaction catalyzed by **1_300_**, **1_350_**, **1_400_**, and **1_500_** at 165 and 185 °C. d) Conversion and selectivity of the continuous catalytic reaction using **1_300_** as a catalyst at 185 °C.

Since the microfibers in **1_300_** possessed meso‐ and macropores, as observed from the FIB–SEM images, N_2_ sorption isotherms were obtained to investigate the pore structures (Figure 6a). As expected, the sorption isotherms showed type H3 hysteresis, corresponding to the aggregation of plate‐like particles with slit‐shaped pores.^[^ ^52^ ^]^ This result also supported the lack of submicroribbon agglomeration in the FIB–SEM images. The Brunauer–Emmett–Teller (BET) surface area of **1_300_** was calculated to be 32.5 m^2^ g^−1^, and this value decreased as the calcination temperature increased (Table S4). Conventional α‐MoO_3_ possesses a very small BET surface area, whereas the surface area of **1_300_** was increased mainly because of the formation of ultrafine nanorods. The IR spectrum of **1_300_** showed broad absorption peaks at 3000–3700 cm^−1^, which were attributed to the stretching vibration of the O─H bonds of the adsorbed water molecules; these peaks were not observed in the spectrum of **1_600_** (Figure S4). A plot of the relative area ratio of the peaks attributed to *ν*(O─H) versus the BET surface area was linear, consistent with the large surface area of **1_300_** (Figure S30a). In addition, the sorption isotherm of **1_300_** showed a sharp increase at low relative pressures, which was attributed to the micropores. To confirm the presence of micropores, STEM was conducted. The STEM image of submicroribbons in **1_300_** projected from the [100] direction showed several dark segments with widths of <1 nm, which were attributed to slit‐shaped micropores (Figure 5j). According to the Horvath–Kawazoe (HK) plot, **1_300_** possessed micropores with a diameter of 0.6 nm (Figure S30b), which was consistent with the thickness of the 2D sheet structure of α‐MoO_3_. The void volume of **1_300_** was determined to be 9.3 mm^3^ g^−1^ (4.4% volume of α‐MoO_3_) on the basis of the micropore volume of 6.0 mL g^−1^ at standard temperature and pressure, consistent with the observation frequency of 24 truncated dark segments per approximately 180 nm (sum of the widths of the nanorods) in the STEM images (Figures 5j and S31). In the FIB–SEM image, cross‐ sections of lamella‐like structures were observed, indicating that the nanorods in **1_300_** in which the (100) plane was the broad face also grew along the [100] direction to form variable‐height ridge‐like structures (Figure S20d,e). Consequently, stacking the variable‐height ridge‐like nanorods along the [010] direction resulted in slit‐shaped boundaries between the nanorods, from which micropores originated (Figure S32). In the synthesis of previously reported functional α‐MoO_3_, the chemical or electrochemical reduction of α‐MoO_3_ afforded hydrogen molybdenum bronze species with high surface areas, while these species often contained reduced phases such as 3D MoO_2_.^[^ ^53^, ^54^, ^55^, ^56^, ^57^ ^]^ The addition of surfactants and the utilization of template structures are also effective methods for synthesizing mesoporous α‐MoO_3_, whereas it remains difficult to prepare microporous α‐MoO_3_;^[^ ^58^, ^59^, ^60^, ^61^, ^62^, ^63^, ^64^, ^65^ ^]^ thus, to the best of our knowledge, **1_300_** is the first reported microporous α‐MoO_3_. The micropore volume was quite low in **1_350_** and **1_400_**, and no micropores were observed in **1_500_** and **1_600_**, indicating that the micropores were also decreased by the reorganization and densification of α‐MoO_3_ crystals.

The high surface area, micropores, and the broad (100) surface of **1_300_** were considered to investigate the catalytic dehydration reaction of 2‐propanol (Figure 6b). The reaction of 2‐propanol in the presence of **1_300_** at 185 °C proceeded efficiently, resulting in >99% conversion (Figures 6c and S33). The selectivity of propylene increased as the reaction temperature increased, whereas that of isopropyl ether decreased because of an increase in the entropic contributions (Figure S34). These two products were obtained via a dehydration reaction. In contrast, the selectivity of acetone, which was an oxidation product, was very low (2%), indicating that the catalytic reaction occurred at acid sites. Because the broad face of **1_300_** was the (100) plane, **1_300_** was expected to possess many coordinatively unsaturated Mo sites where water molecules could coordinate (Figure S4).^[^ ^66^ ^]^ The ESR spectrum of **1_300_** after vacuum drying at 200 °C for 10 min showed that the intensity of the signal assignable to four‐coordinate Mo^5+^ centers increased (Figure S35a).^[^ ^51^ ^]^ By addition of vapor of 2‐propanol, the intensity of the same signal decreased, indicating the coordination of 2‐propanol at the coordinatively unsaturated Mo sites (Figure S35b,c). Therefore, the catalytic active sites of **1_300_** were determined to be Lewis acid sites. As expected, the reaction did not proceed in the presence of **1_600_** because of the (010) plane as a broad face with the low surface area and the absence of the coordinatively unsaturated Mo sites like conventional α‐MoO_3_ (Table S4; Figures 6a, S1, S16e, and S26f). Furthermore, **1_300_** was hypothesized to be a stable catalyst because the broad face of the (100) plane was retained during calcination for 6 h, as determined from the SAED patterns of **1_300_^10^** and **1_300_**; thus, a continuous catalytic reaction was examined. As a result, the catalytic activity of **1_300_** can be maintained for 24 h, keeping high conversion and selectivity (Figure 6d). The XRD pattern of the catalyst after the reaction showed broad diffraction peaks that were attributed to α‐MoO_3_ (Figure S36). The diffraction peak attributed to the 110 reflection was still strong, indicating that the broad (100) surface was retained even after the reaction. The size of crystallites was calculated by means of Scherrer's equation using the FWHM of the 020 reflection (17.1 nm), and this value was slightly larger than that of **1_300_** (15.3 nm) but still much smaller than that of **1_350_** (26.0 nm), strongly supporting the high thermal stability of **1_300_**.

## Conclusion

In this study, we successfully demonstrated a structure‐preserving dimensionality‐increasing strategy to synthesize microporous α‐MoO_3_ in which the (100) plane was the broad face. In situ‐formed 0D [Mo_2_O_5_(H_2_O)_6_]^2+^ ({Mo_2_}) as a minimum structural motif of 2D α‐MoO_3_ was polymerized efficiently to form 1D [Mo_2_O_6_(DMF)]*_n_* (**1**) by adding capping DMF molecules at both ends of {Mo_2_}. Calcination of 1D **1** at 300 °C (**1_300_**) induced the removal of DMF and the assembly of subnanofibers within a microfiber in **1** to afford 2D α‐MoO_3_. Although it has been quite difficult to control the intermediate structures generated by conventional methods for the synthesis of α‐MoO_3_ from 0D precursors, the unique dimensionalities of the intermediate compounds in this study increased in a stepwise manner, and the structures of the precursors were preserved (0D {Mo_2_} → 1D **1** → 2D α‐MoO_3_). Below the calcination temperature of 350 °C, the stacking reaction of the subnanofibers along the [010] direction was preferential over the condensation reaction of the subnanofibers along the [100] direction, resulting in the formation of ultrafine nanorods of α‐MoO_3_ in which the (100) plane was the broad face. As the fibrous crystal morphology was retained during calcination, the hierarchical structure of **1_300_** (microfibers ⊃ submicroribbons ⊃ nanorods) was formed. The unique surface structure of **1_300_**, including micropores and a large specific surface area, made **1_300_** a high‐performance active acid catalyst.

It is our hope that the facile gram‐scale synthesis of α‐MoO_3_ in which the (100) plane is the broad face developed in this study will open a new horizon for achieving unique catalytic, magnetic, and electrode materials with excellent properties. Additionally, it is expected that this structure‐preserving dimensionality‐increasing strategy will allow the synthesis of other types of metal oxides, including metastable 2D and 3D structures, the design and control of defects and surface structures at the atomic level, and clarification of synthesis–structure correlations.

## Conflict of Interests

The authors declare no conflict of interest.

## Supporting information



Supporting Information

## Data Availability

The data that support the findings of this study are available in the Supporting Information of this article.
