# Supplementary material for: A Structure‐Preserving, Dimensionality‐Increasing Strategy for the Stepwise Synthesis of Microporous α‐MoO3 with a Broad (100) Surface
Source: Angew Chem Int Ed Engl. 2025 Jun 23;64(33):e202506758. doi: 10.1002/anie.202506758 (PMC12338404; doi:10.1002/anie.202506758)
Supplement: Supplementary file 1 — Supporting Information [file ANIE-64-e202506758-s001.pdf]

*Supporting Information for*

# A Structure-Preserving, Dimensionality-Increasing Strategy for the Stepwise Synthesis of Microporous $\alpha$-$MoO_3$ with a Broad (100) Surface

Takuo Minato,*[a] Misato Miyamoto,[a] Satoshi Ishikawa,†[b] Norihito Hiyoshi,[c] Makoto Maeda,[d] Kenji Komaguchi,[a] Masahiro Sadakane[a]

[a] Department of Applied Chemistry, Graduate School of Advanced Science and Engineering, Hiroshima University, 1-4-1 Kagamiyama, Higashi-Hiroshima, Hiroshima, 739-8527, Japan.

[b] Department of Material and Life Chemistry, Faculty of Engineering, Kanagawa University, 3-27-1 Rokkakubashi, Kanagawa-ku, Yokohama, Kanagawa, 221-8686, Japan.

[c] Research Institute for Chemical Process Technology, National Institute of Advanced Industrial Science and Technology, 4-2-1 Nigatake, Miyagino-ku, Sendai, Miyagi, 983-8551, Japan.

[d] Natural Science Center for Basic Research and Development, Hiroshima University, 1-3-1 Kagamiyama, Higashi-Hiroshima, Hiroshima, 739-8530, Japan.

**Correspondence**

Dr. Takuo Minato, Department of Applied Chemistry, Graduate School of Advanced Science and Engineering, Hiroshima University, 1-4-1 Kagamiyama, Higashi-Hiroshima, Hiroshima, 739-8527, Japan

Tel: +81-82-424-7605

E-mail: tminato@hiroshima-u.ac.jp

† Present address: Materials and Structures Laboratory, Institute of Integrated Research, Institute of Science Tokyo, 4259 Nagatsuta-cho, Midori-ku, Yokohama, Kanagawa, 226-8501, Japan.

**Experimental Section**

**Materials and Instruments**

$Na_2MoO_4 \cdot 2H_2O$ (FUJIFILM Wako pure chemical), nitric acid (Kanto chemical), DMF (Kanto chemical), *N*-methylformamide (FUJIFILM Wako pure chemical), formamide (FUJIFILM Wako pure chemical), isobutyraldehyde (TCI), and $MoO_3$ as conventional α-$MoO_3$ (FUJIFILM Wako pure chemical) were used as received. IR spectra were measured on a JASCO FT/IR-4X spectrometer using KBr disks. Raman spectra were measured on a JASCO RMP-510 spectrometer using green laser (532 nm). Powder X-ray diffraction (XRD) patterns were collected using a Bruker D2 Phaser with Cu Kα radiation ($\lambda$ = 1.54184 Å, 30 kV, 10 mA) equipped with a 1D Lynxeye detector. Diffuse reflectance UV/vis spectra were measured on a Shimadzu UV-3600 Plus spectrometer using barium sulfate as a reference. Thermogravimetric and differential thermal analyses (TG-DTA) were performed on a Hitachi SII TG/DTA7300 analyzer. Temperature programmed desorption (TPD) mass data was measured on a Microtrac BELCAT II with BELMASS II spectrometer. $N_2$ sorption isotherms were measured on a Microtrac BELSORP MAX. Prior to the sorption measurements, accurately weighted solid samples (ca. 0.5 g) were treated in vacuum at 100 °C for 6 h to remove adsorbed water molecules. Sorption equilibrium was judged by the following criteria: ±0.3% of pressure changes in 5 min. The surface area was determined by a multipoint Brunauer−Emmett−Teller (BET) method using the adsorption range of $0.05 \leq P/P_0 \leq 0.30$. Electron spin resonance (ESR) spectra were measured on a JEOL JES-RE1X spectrometer (X-band) at ca. 298, 77, and 4 K. Microwave power was typically set to 1.0 mW, and 0.1 mW or less was used for the spectrum measured at 4 K. The *g* values were determined by the line shape simulation for the ESR spectra measured with a Bruker ELEXSYS E500 spectrometer (X-band) using $Cr^{3+}$ ($g$ = 1.97984) within MgO as a standard sample. Simulation of the spectrum was conducted by assuming three anisotropic *g* values although the observed spectrum was presumably composed of several signals assignable to $Mo^{5+}$ centers with different coordination environment. Scanning electron microscopy (SEM) images were obtained using a Hitachi High-Tech S-4800 instrument at an acceleration voltage of 15 kV. Samples were directly deposited on a conductive carbon tape. Transmission electron microscopy (TEM) images were obtained using a JEOL JEM-2011 or JEM-F200 instrument at an acceleration voltage of 200 kV. Samples were dispersed in ethanol and sonicated for 5 min before deposition on a carbon-coated copper grid for TEM observation. Scanning transmission electron microscopy (STEM) images were obtained using a JEOL ARM-200F electron microscope at an acceleration voltage of 200 kV with a Cs corrector. Focused ion beam (FIB) SEM images were obtained using a Thermo Scientific (FEI) Helios G4 UC instrument at an acceleration voltage of 15 kV. Samples were directly deposited on a conductive carbon tape. Before the FIB etching process, Pt was deposited on a microfiber (30 kV, 0.77 nA). The Ga ion beam at tilt angle of 52° to the electron beam was used for rough milling (30 kV, 83 pA) and fine milling (30 kV, 33 pA). Inductivity coupled plasma atomic emission spectroscopy (ICP-AES) analyses for Mo were performed with Thermo Fisher Scientific iCAP 6000. Elemental analyses for C, H, and N were performed at the Natural Science Center for Basic Research and Development, Hiroshima University and the Service Center of the Elemental Analysis of Organic Compounds, Faculty of Science, Kyushu University. Electric furnace (FO410, Yamato) was used for the calcination of **1**. Temperature control was programmed with the ramp rate of 5 °C/min from room temperature to designated temperature *n* °C for $\mathbf{1}_{n}$ and $\mathbf{1}_{300}^{10}$. The samples $\mathbf{1}_{300}^{6}$, $\mathbf{1}_{300}^{8}$, and $\mathbf{1}_{300}^{10}$ were prepared by inserting **1** into preheated furnace at 300°C.

**Catalytic dehydration of 2-propanol**

Catalytic dehydration reaction of 2-propanol was carried out using a conventional vertical flow system with a Pyrex tubular reactor at ambient pressure. The catalyst $\mathbf{1}_n$ (0.1 g) was diluted with 2.0–2.5 g of SiC, and the mixture was set in the tubular reactor. The reactor was heated at a ramp rate of 10°C min$^{-1}$ to 200°C under $N_2$ flow (20 mL min$^{-1}$). The temperature in the catalyst bed was measured with a thermocouple inserted in the middle of the catalyst zone. When the temperature reached 200°C, a reactant gas with the composition of 2-propanol/$O_2$/$N_2$ = 1.1–1.5/4.0/20 mL min$^{-1}$ was flowed. 2-propanol was supplied by $N_2$ bubbling to liquid 2-propanol solution held at room temperature. After the reaction at 200°C, the temperature was decreased to 180, 160, and 140°C with analysis of the reactant and product gases at each reaction temperature. Reactants and products were analyzed with three on-line gas chromatographs. $O_2$, $N_2$, and CO were detected by a thermal conductivity detector using Molecular Sieve 5A. $CO_2$, $H_2O$, and propylene were analyzed by a thermal conductivity detector using Gaskuropak 54. 2-propanol, acetone, and isopropyl ether were analyzed by a frame ionization detector using Porapack QS. Blank runs showed that no reaction took place without catalysts under the experimental condition used in this study. The carbon balance in the reactions was 98−101%; thus, the product selectivity was calculated on a product amount basis.

**X-ray Crystallography**

Diffraction measurements were made on a Bruker SMART APEX2 CCD detector with Mo $K_\alpha$ radiation ($\lambda$ = 0.71073 Å, 50 kV, 24 mA) monochromated by layered confocal mirrors at 123 K. Data reduction, integration, scaling, and space group determination were carried out using the Bruker APEX3 suite.[S1] The structural analyses were performed using WinGX.[S2] All structures were solved by SHELXT-2014/5[S3] and refined by SHELXL-2018/3[S4]. All atoms were refined anisotropically. CCDC-2427315 (**1**) contains the supplementary crystallographic data for this paper. The data can be obtained free of charge via https://www.ccdc.cam.ac.uk/structures/.

**Bond valence sum (BVS) calculation**

The BVS values were calculated by the expression for the variation of the length $r_{ij}$ of a bond between two atoms $i$ and $j$ in observed crystal with valence $V_i$.

$$V_i = \sum_j \exp\left(\frac{r'_0 - r_{ij}}{B}\right)$$

where $B$ is constant equal to 0.37 Å, $r'_0$ is bond valence parameter for a given atom pair.[S5]

**Synthesis and characterization of $Mo_2O_6(C_3H_7NO)$ (1)**

$Na_2MoO_4 \cdot 2H_2O$ (10.0 g, 41.3 mmol) was dissolved in 1.0 L of water, and then concentrated nitric acid (13.0 M, 20.0 mL) was added to the resulting solution. The mixture was incubated at room temperature (approximately 25°C) in a light-protected environment using the DMF vapor diffusion method for crystallization (Figure S37). Colorless fibrous single crystals were formed within few days. After 4 weeks, crystals of **1** were isolated by filtration, washed with an excess amount of water, and dried in vacuo (7.1 g, 95% yield based on Mo). These crystals were then calcinated at various temperature to prepare $\mathbf{1}_n$. The reproducibility of gram-scale synthesis was confirmed more than 10 times (average 95% yield based on Mo). The larger single crystals of **1** suitable for X-ray crystallographic analysis could

be obtained when crystallizing in a synthesis solution diluted approximately tenfold (Figure S38). When employing a more concentrated synthesis solution (200 mg of $Na_2MoO_4 \cdot 2H_2O$ in 2.0 mL of 1.0 M $HNO_3$ solution), the crystallization period could be shortened (within a week, 97% yield based on Mo), where obtained crystals were characterized as **1** by powder XRD (Figure S7d). In this study, the Raman spectrum of this concentrated solution was measured to clearly observe the peak at 955 $cm^{-1}$. Note that this crystalline material possessed not fibrous but hemispherical crystal morphology due to the rapid crystallization process, which was also supported by the simulated XRD patterns (Figure S7). IR (KBr pellet): 2988, 2950, 2925, 2857, 2810, 1664, 1500, 1427, 1373, 1248, 1119, 1059, 958, 932, 907, 791, 697, 557, 421 $cm^{-1}$; elemental analysis calcd (%) for $C_3H_7Mo_2NO_7$: C 9.98, H 1.95, N 3.88, Mo 53.16; found: C 9.93, H 2.05, N 3.80, Mo 52.98.

**Table S1**. Crystallographic data for **1**.

| | **1** |
|---|---|
| Formula | $C_3H_7Mo_2NO_7$ |
| Fw (g mol$^{-1}$) | 360.98 |
| Crystal system | Monoclinic |
| Space group | $P2_1/c$ (#14) |
| $a$ (Å) | 3.7268(12) |
| $b$ (Å) | 22.018(7) |
| $c$ (Å) | 10.177(3) |
| $\alpha$ (deg) | 90 |
| $\beta$ (deg) | 94.194(4) |
| $\gamma$ (deg) | 90 |
| $V$ (Å$^3$) | 832.8(5) |
| $Z$ | 4 |
| Temp (K) | 123(2) |
| $\rho_{calcd}$ (g cm$^{-3}$) | 2.879 |
| GOF | 1.058 |
| $R_1$[a] [I>2σ($I$)] | 0.0409 |
| $wR_2$[a] | 0.1047 |

[a] $R_1 = \Sigma||F_o| - |F_c||/\Sigma|F_o|$, $wR_2 = \{\Sigma[w(F_o^2 - F_c^2)]/\Sigma[w(F_o^2)^2]\}^{1/2}$.

**Table S2**. BVS values of oxygen and metal atoms of **1**.

| | |
|---|---|
| O1 | 1.894 |
| O2 | 1.754 |
| O3 | 1.956 |
| O4 | 2.117 |
| O5 | 2.074 |
| O6 | 1.807 |
| O7 | 1.769 |
| Mo1 | 6.005 |
| Mo2 | 5.922 |

**Table S3**. Distances between C and O atoms in **1**.

| | |
|---|---|
| C1···O2 | 3.169 Å |
| C1···O2 | 3.453 Å |
| C2···O6 | 3.477 Å |
| C2···O6 | 3.543 Å |
| C2···O6 | 3.545 Å |
| C2···O6 | 3.857 Å |
| C2···O7 | 3.153 Å |
| C2···O7 | 3.507 Å |
| C3···O2 | 3.453 Å |
| C3···O2 | 3.785 Å |
| C3···O7 | 3.050 Å |
| C3···O7 | 3.416 Å |

**Table S4**. BET surface area of $\mathbf{1}_{n}$.

| | |
|---|---|
| $\mathbf{1}_{300}$ | 32.5 $m^2\ g^{-1}$ |
| $\mathbf{1}_{350}$ | 20.2 $m^2\ g^{-1}$ |
| $\mathbf{1}_{400}$ | 8.2 $m^2\ g^{-1}$ |
| $\mathbf{1}_{500}$ | 1.1 $m^2\ g^{-1}$ |
| $\mathbf{1}_{600}$ | 0.4 $m^2\ g^{-1}$ |

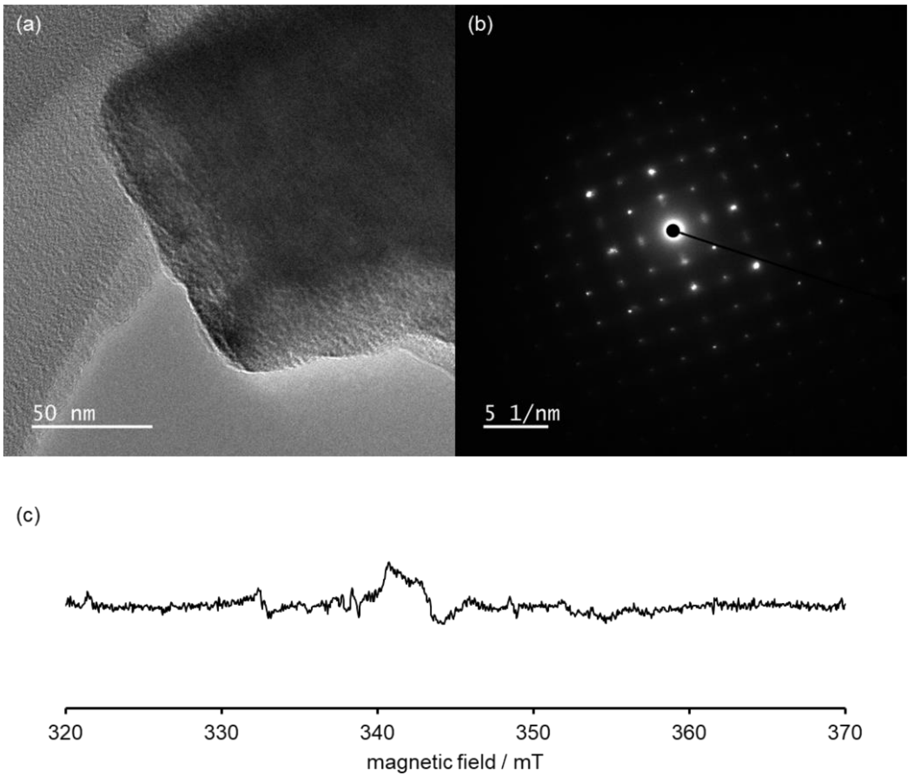


**Figure S1.** (a) TEM image, (b) corresponding SAED pattern, and (c) ESR spectrum at 77 K of conventional plate-like crystals of $\alpha$-$MoO_3$ (FUJIFILM Wako pure chemical). The SAED pattern showed the diffraction spots that were attributed to the pattern projected from the [010] direction, indicating that the observed surface of plate-like crystals was (010) plane. The ESR spectrum showed no signals assignable to the $Mo^{5+}$ centers, indicating the absence of oxygen defects in the conventional $\alpha$-$MoO_3$.

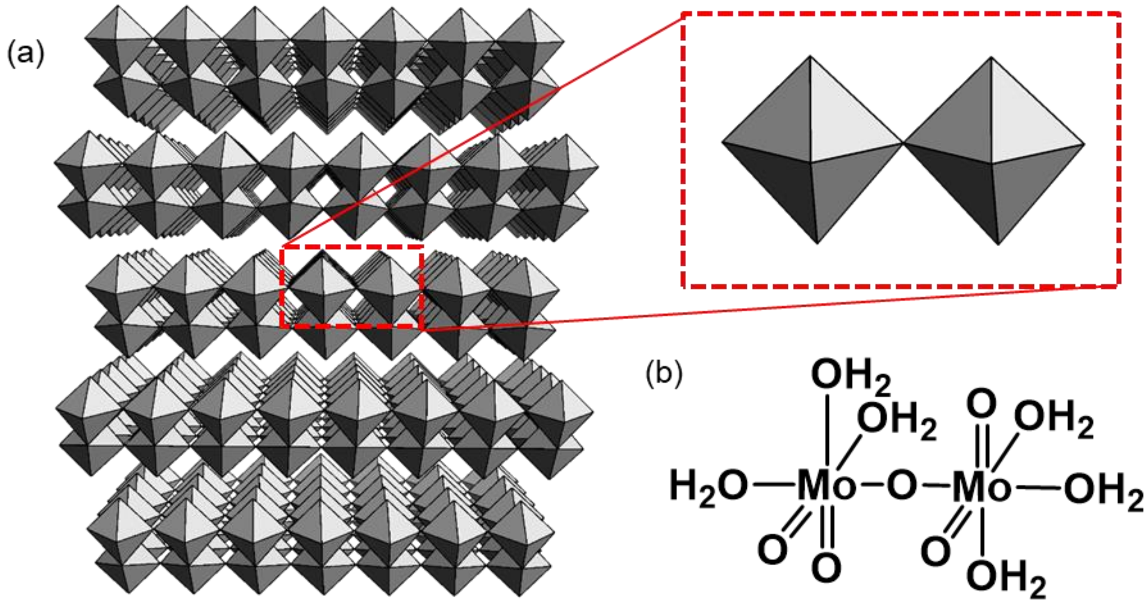


**Figure S2.** (a) Crystal structure of 2D α-$MoO_3$ along the [010] direction and di-nuclear Mo species as a structural motif of α-$MoO_3$. (b) Proposed structural model of $[Mo_2O_5(H_2O)_6]^{2+}$ species ($\{Mo_2\}$).

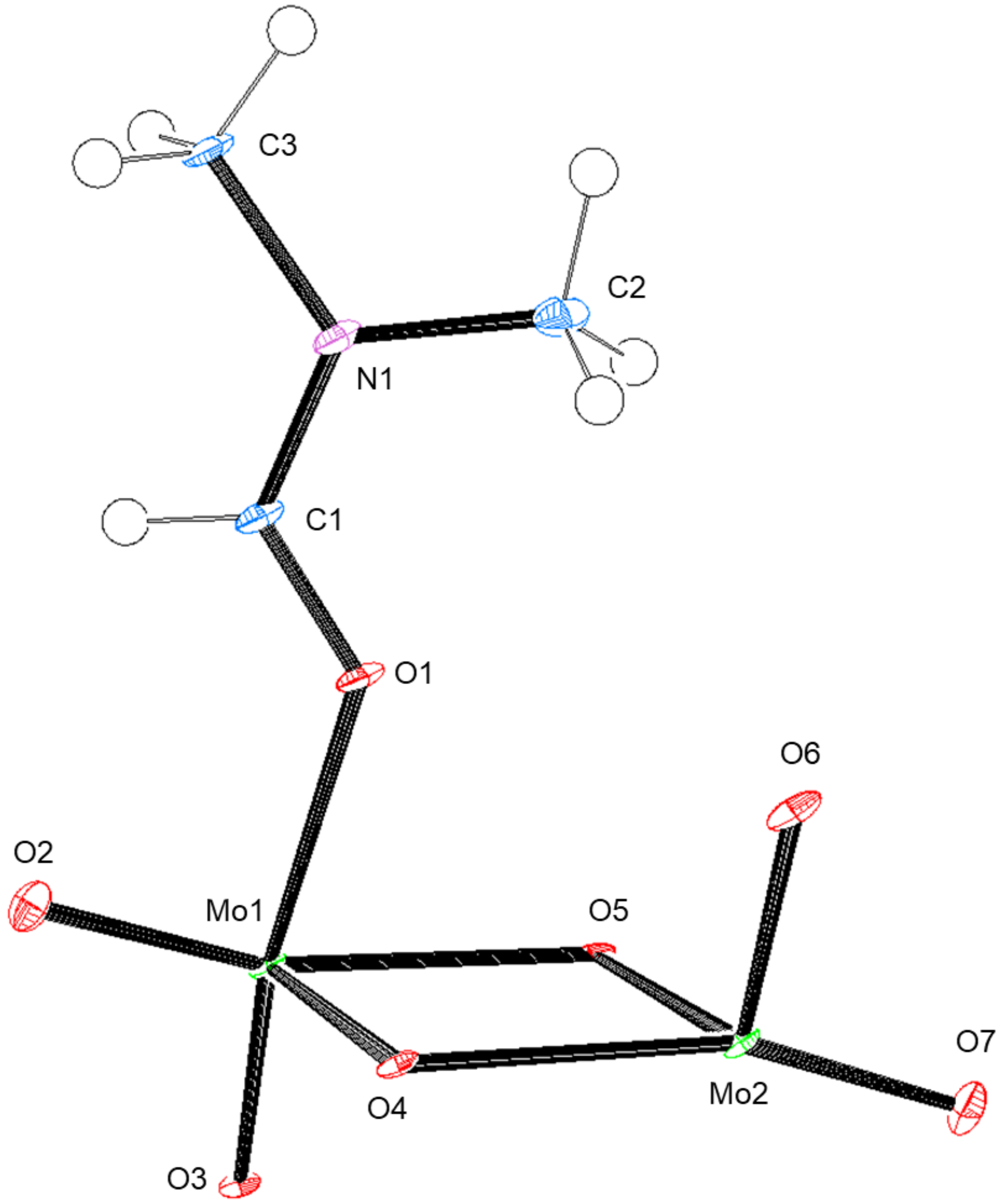


**Figure S3.** ORTEP representations of **1** with thermal ellipsoids drawn at the 50% probability level. The atoms are represented by octant shading spheres; O: red, Mo: green, C: blue, N: pink, H: white.

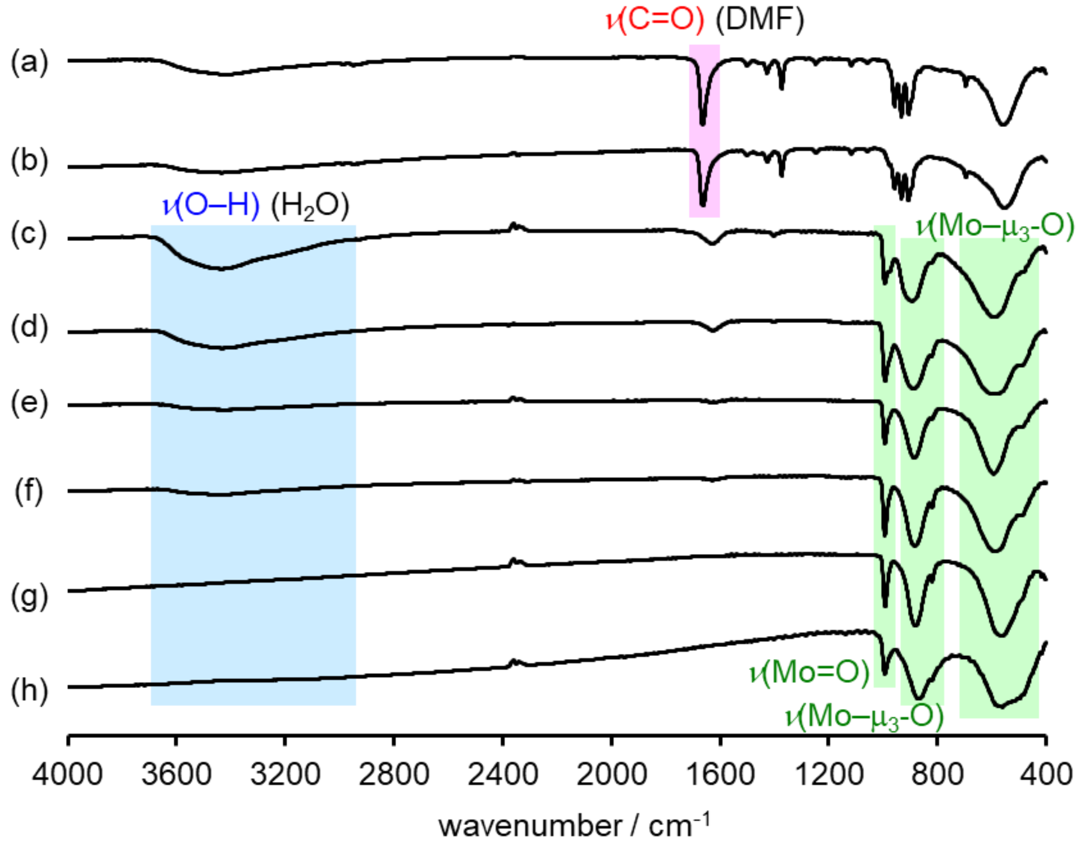


**Figure S4.** IR spectra of (a) **1**, (b) $\mathbf{1}_{200}$, (c) $\mathbf{1}_{250}$, (d) $\mathbf{1}_{300}$, (e) $\mathbf{1}_{350}$, (f) $\mathbf{1}_{400}$, (g) $\mathbf{1}_{500}$, and (h) $\mathbf{1}_{600}$.

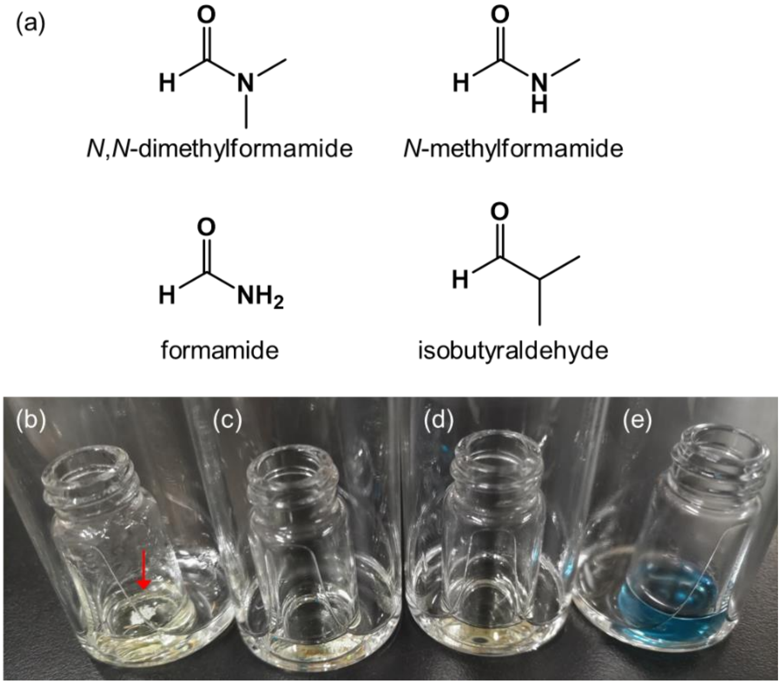


**Figure S5.** (a) Structures of capping organic molecules used in this study. Photo of crystallization solutions of {$Mo_2$} after 3 days using (b) DMF, (c) *N*-methylformamide, (d) formamide, and (e) isobutyraldehyde as capping organic molecules. Red arrow represents the formation of crystals **1**. The color of the solution turned to blue when using isobutyraldehyde, indicating the intervalence charge transfer of $Mo^{6+}$–O–$Mo^{5+}$ due to the reduction of $Mo^{6+}$ by isobutyraldehyde.

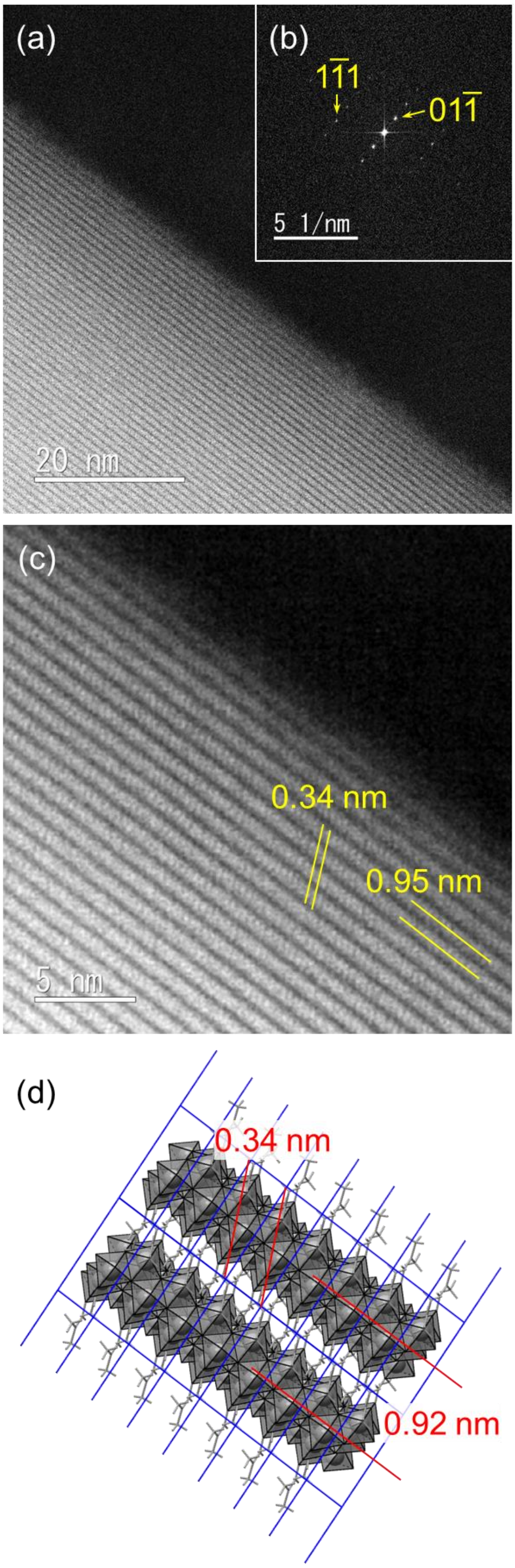


**Figure S6.** (a) STEM image of **1** projected from the [011] direction. (b) SAED pattern of the image a. Enlarged image of a after an image processing. (d) Crystal structure of **1** projected from the [011] direction. Blue lines represent unit cells.

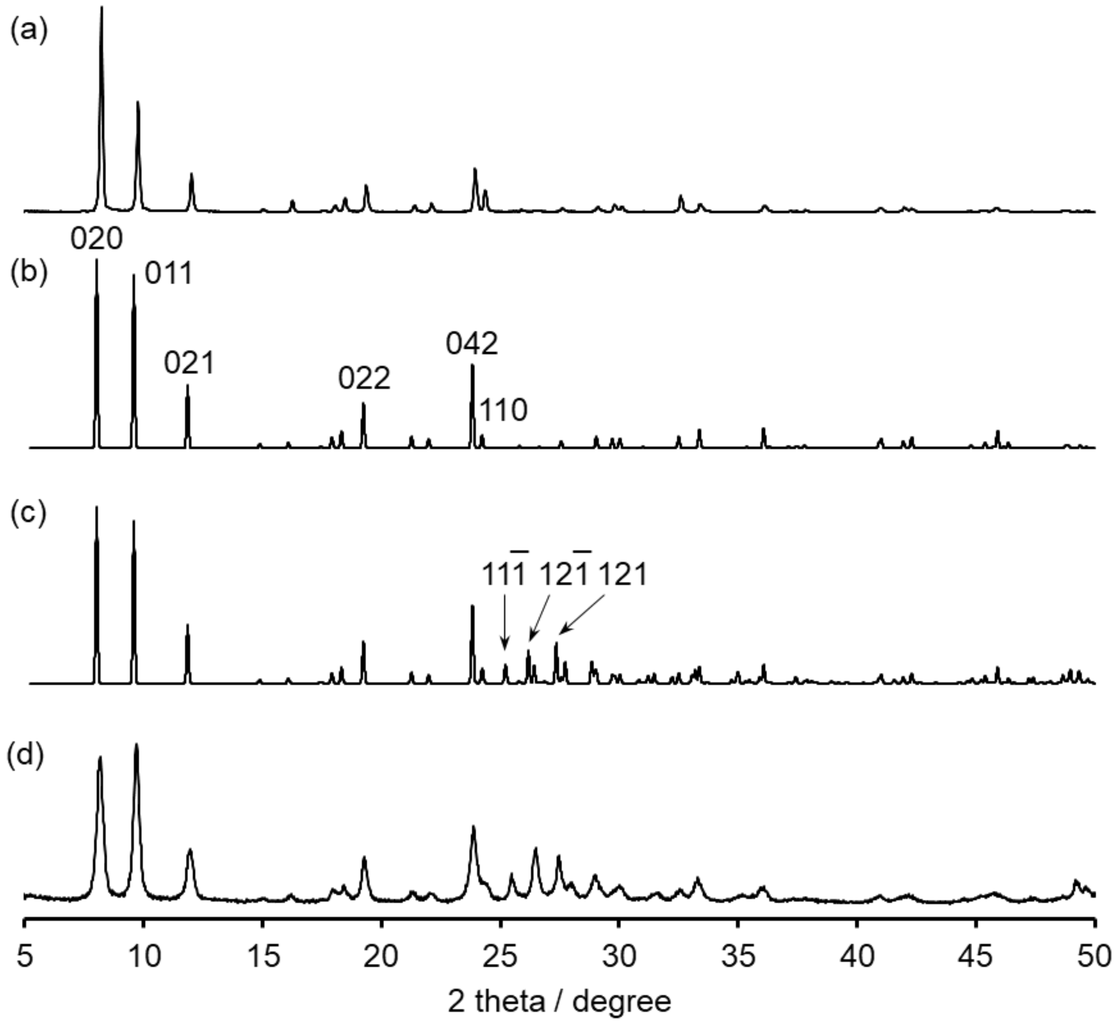


**Figure S7.** (a) Measured and (b, c) simulated XRD patterns of **1**. Needle-type crystal orientation along the [100] direction was considered in the simulation b with the alignment factor of 0.9. No crystal orientation was considered in the simulation c. (d) XRD pattern of **1** synthesized by using the concentrated synthesis solution. See the Experimental Section for the details.

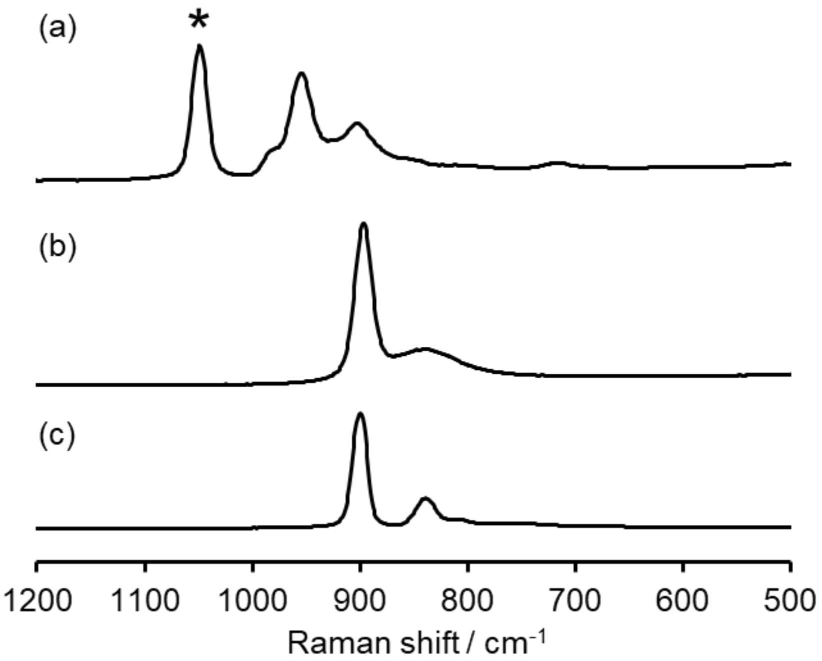


**Figure S8.** Raman spectra of (a) $Na_2MoO_4 \cdot 2H_2O$ (0.4 M) in 1 M $HNO_3$ aqueous solution, (b) $Na_2MoO_4 \cdot 2H_2O$ (0.4 M) in water, and (c) $Na_2MoO_4 \cdot 2H_2O$ (solid). The peak with asterisk is assignable to $NO_3^-$. See the Experimental Section for preparing the solution in this measurement.

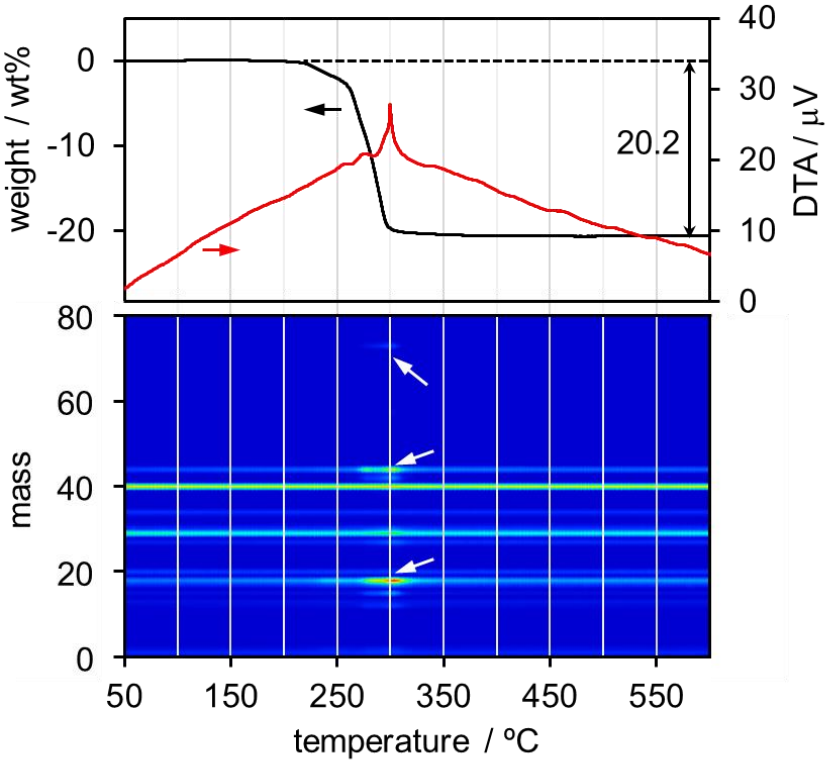


**Figure S9.** Combined TG-DTA and TPD mass data of **1** under air with the ramp rate of 2°C min$^{-1}$. In the TPD mass data, strong signals at *m/z* 14 (N), 16 (O), 28 ($N_2$), and 32 ($O_2$) were omitted for clarity. White arrows represent DMF and fragmentated ion signals at *m/z* 73, 44, and 18, assignable to $[C_3H_7NO]^+$, $[CH_2NO]^+$, and $[NH_4]^+$, respectively.[S6]

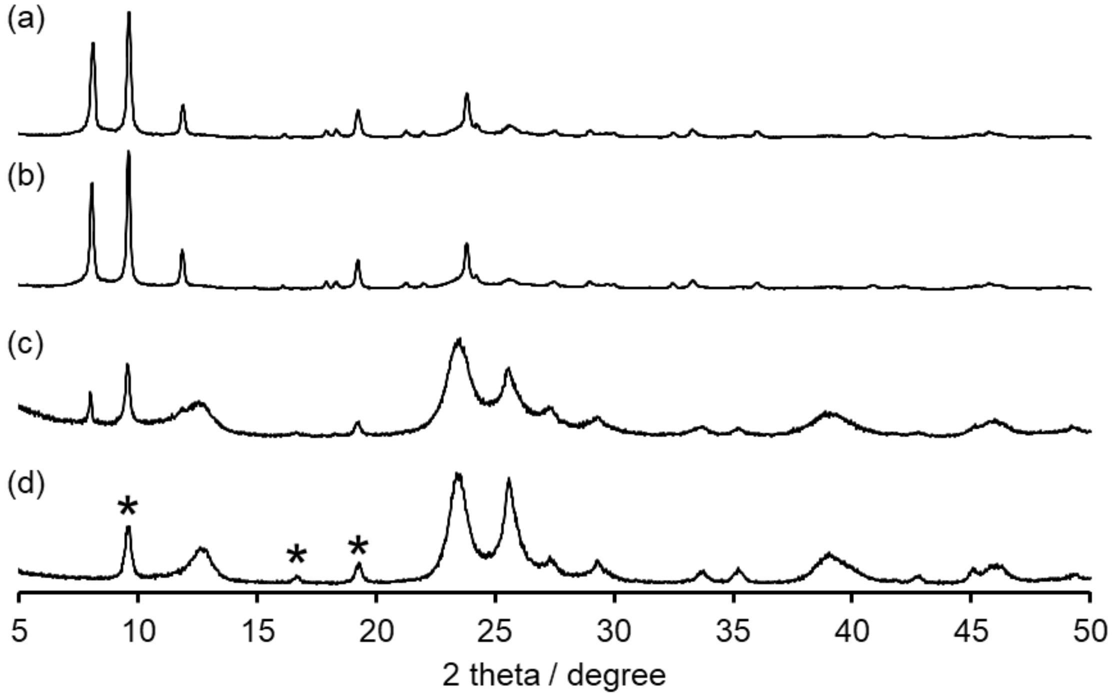


**Figure S10.** XRD patterns of (a) $\mathbf{1_{210}}$, (b) $\mathbf{1_{215}}$, (c) $\mathbf{1_{220}}$, and (d) $\mathbf{1_{225}}$ in the structural transformation from **1** into α-$MoO_3$. Diffraction peaks with asterisks in the pattern d are presumably assignable to h-$MoO_3$.

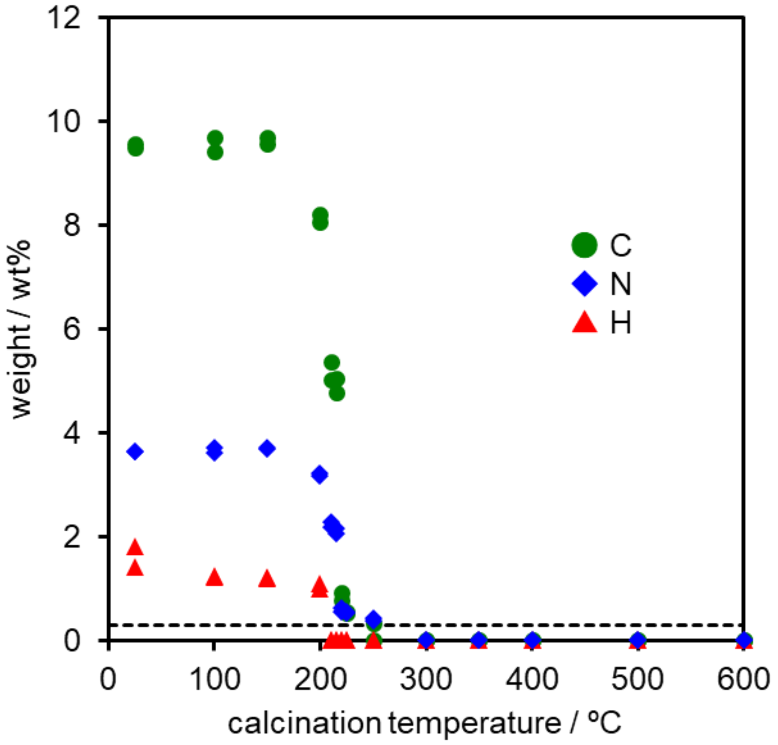


**Figure S11.** Plots of the weights of $\mathbf{1}_n$ for C, H, and N determined by the elemental analyses. Each sample was analyzed twice. Dashed line represents the detection limit.

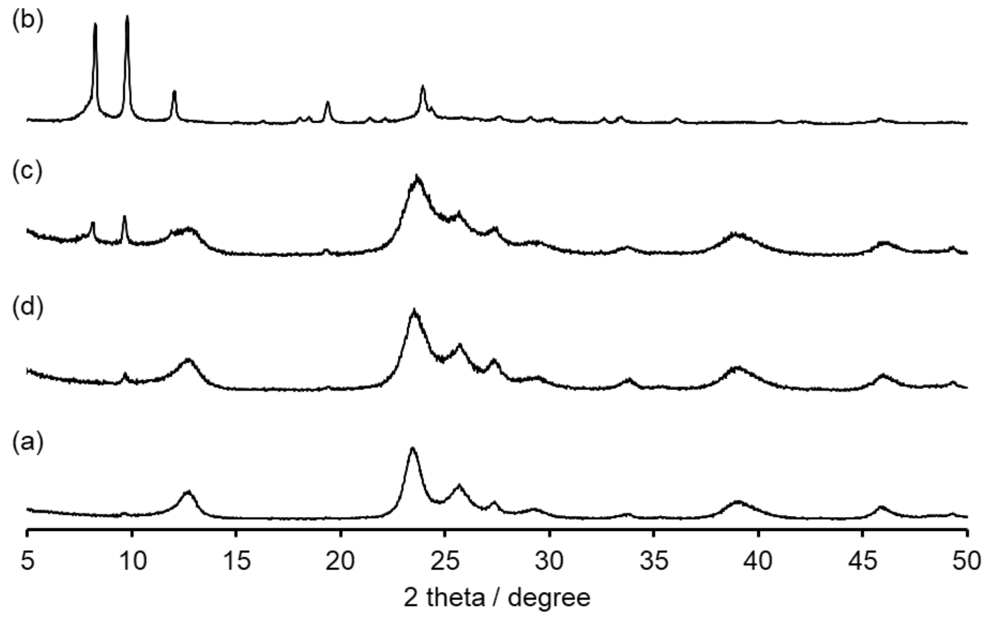


**Figure S12.** XRD patterns of (a) $\mathbf{1}_{300}^{10}$ prepared by temperature-programmed calcination (ramp rate of 5 °C/min, keeping 300 °C for 10 min, and air cooling to room temperature), (b) $\mathbf{1}_{300}^{6}$, (c) $\mathbf{1}_{300}^{8}$, and (d) $\mathbf{1}_{300}^{10}$ prepared by inserting **1** into preheated furnace at 300°C and taking out the samples in 6, 8, and 10 min, respectively.

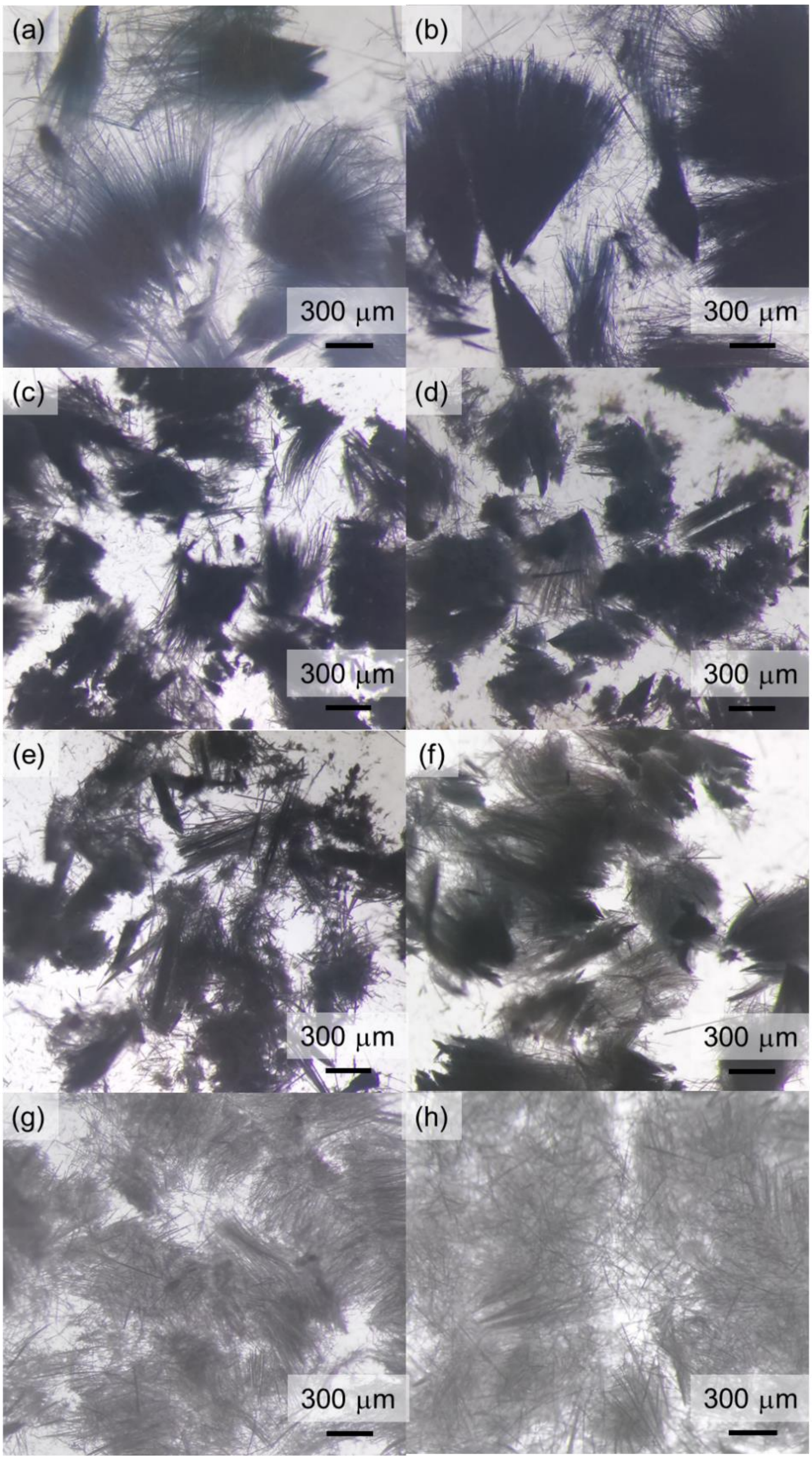


**Figure S13.** Optical microscopy images of (a) $\mathbf{1}_{150}$, (b) $\mathbf{1}_{200}$, (c) $\mathbf{1}_{250}$, (d) $\mathbf{1}_{300}$, (e) $\mathbf{1}_{350}$, (f) $\mathbf{1}_{400}$, (g) $\mathbf{1}_{500}$, and (h) $\mathbf{1}_{600}$.

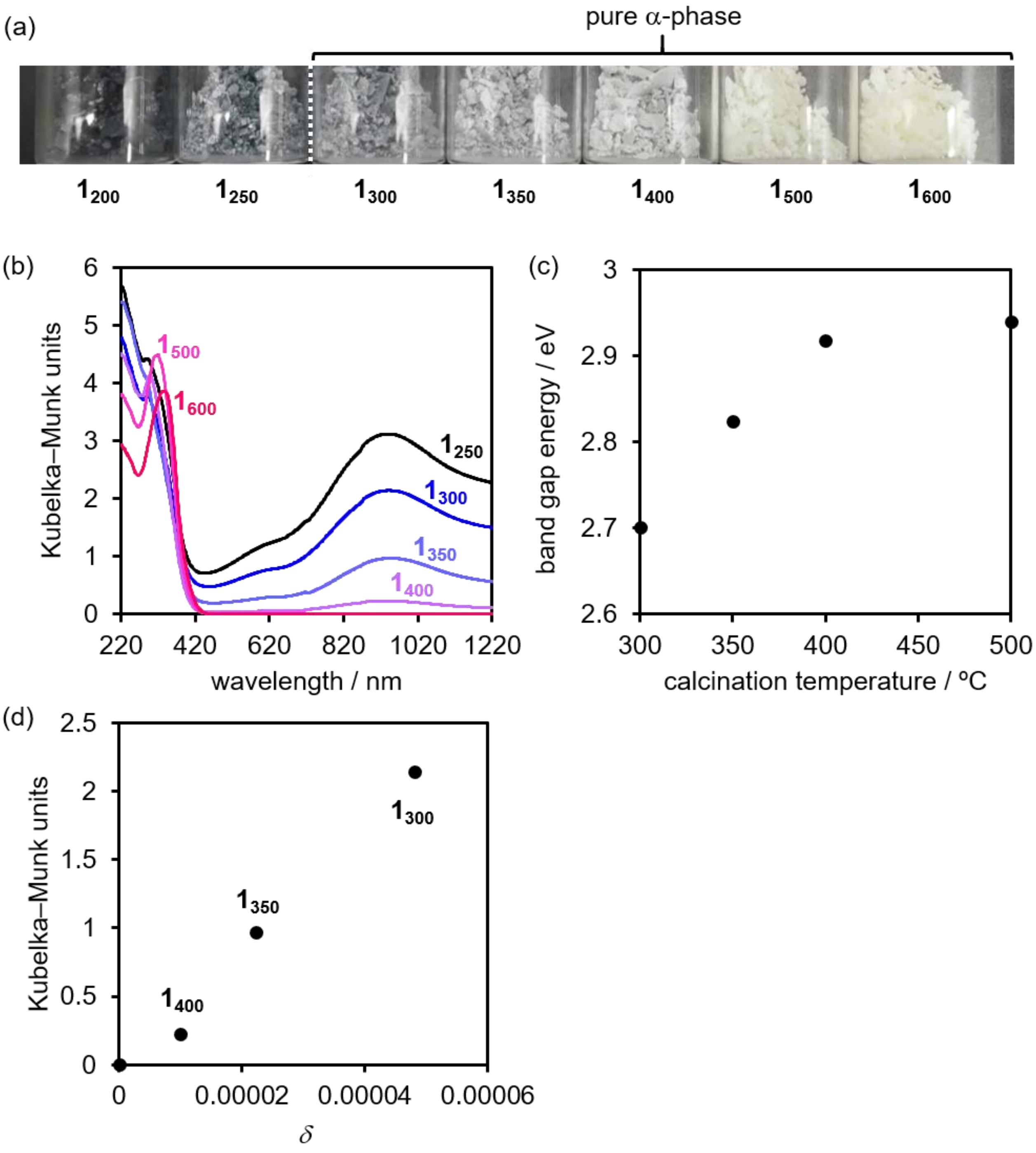


**Figure S14.** (a) Photos of the crystalline powder samples of $\mathbf{1}_n$. (b) Diffuse reflectance UV/Vis spectra of $\mathbf{1}_{250\text{–}600}$. (c) Plots of band gap energies of $\mathbf{1}_n$ versus calcination temperatures. (d) Plots of Kubelka–Munk units of $\mathbf{1}_n$ at 940 nm versus stoichiometry deviation $\delta$ in $MoO_{(3-\delta)}$.

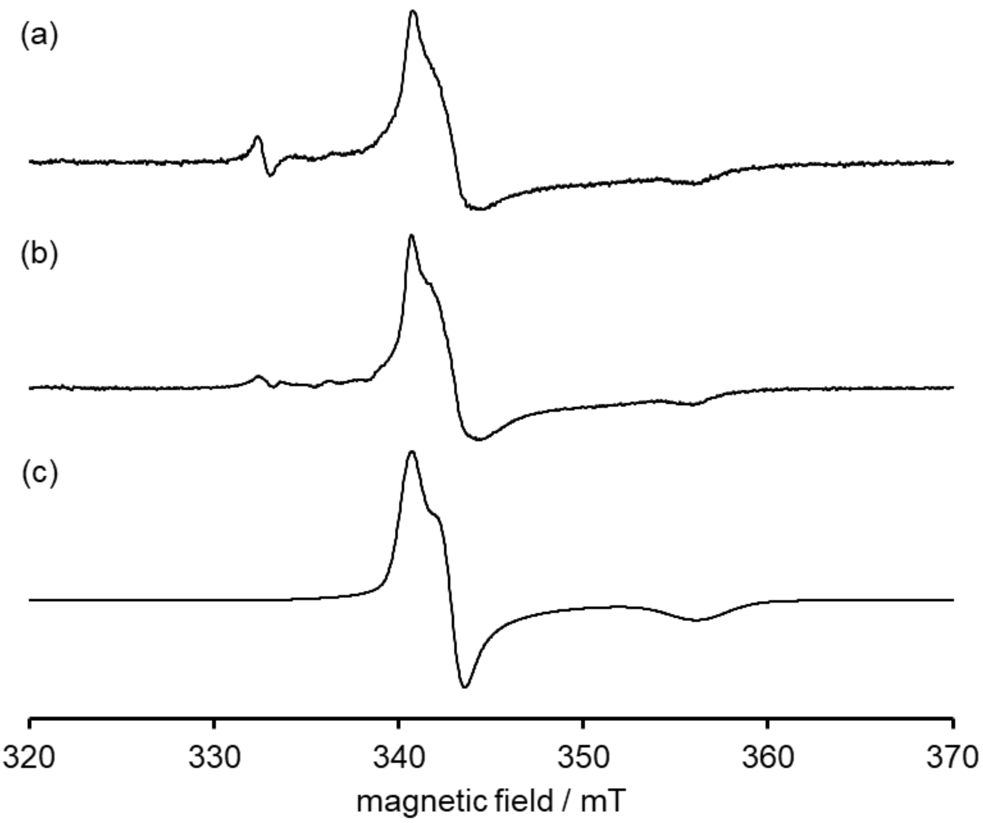


**Figure S15.** ESR spectra of $\mathbf{1}_{300}$ recorded at (a) 77 K and (b) 4K. (c) Simulated ESR spectrum of the observed spectrum b with $g_1$ = 1.9626, $g_2$ = 1.9498, $g_3$ = 1.8764.

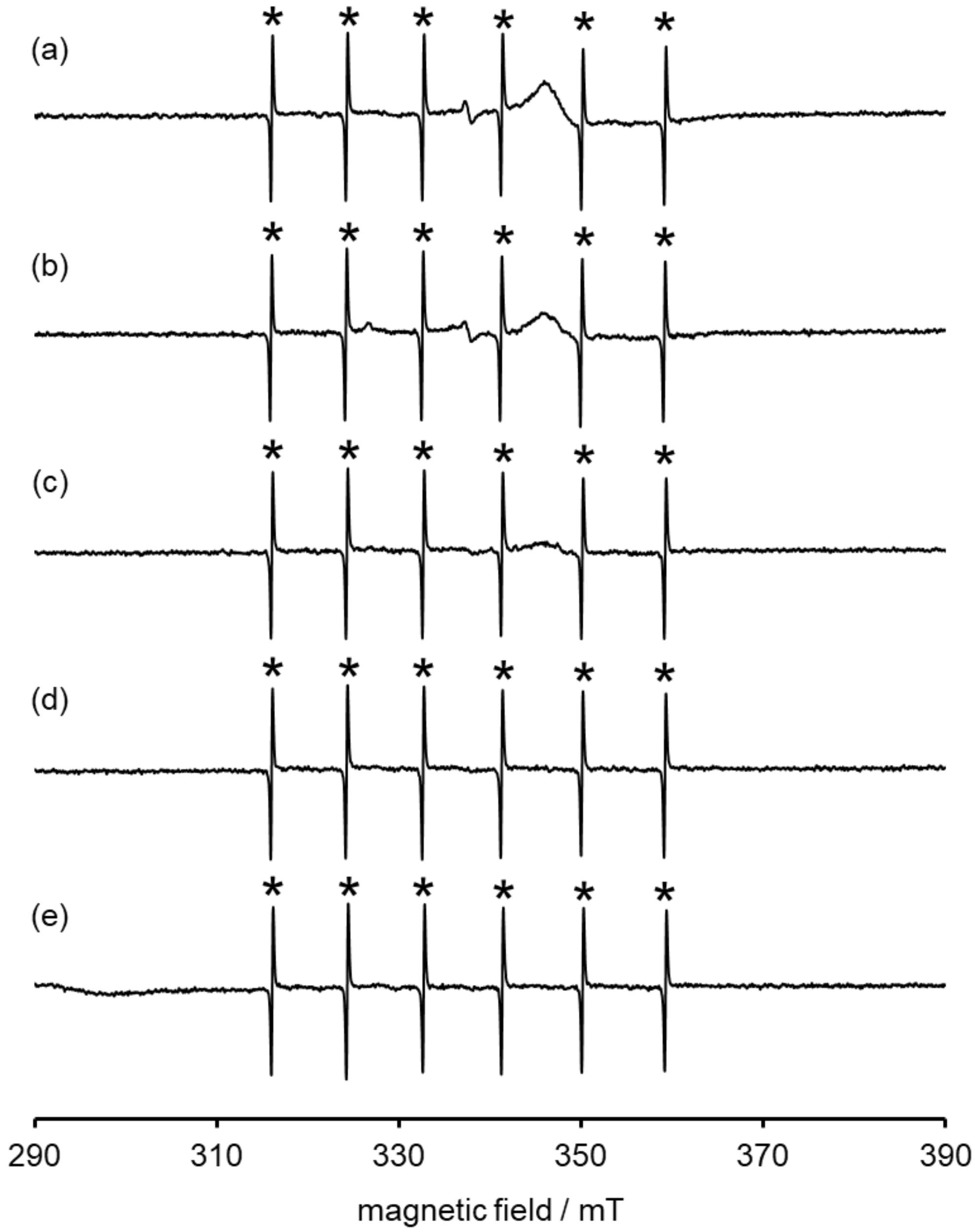


**Figure S16.** ESR spectra of (a) $\mathbf{1}_{300}$, (b) $\mathbf{1}_{350}$, (c) $\mathbf{1}_{400}$, (d) $\mathbf{1}_{500}$, and (e) $\mathbf{1}_{600}$ at room temperature (ca. 25°C). Six signals with asterisks were assignable to the hyperfine structure of $Mn^{2+}$ within MgO as a standard sample.

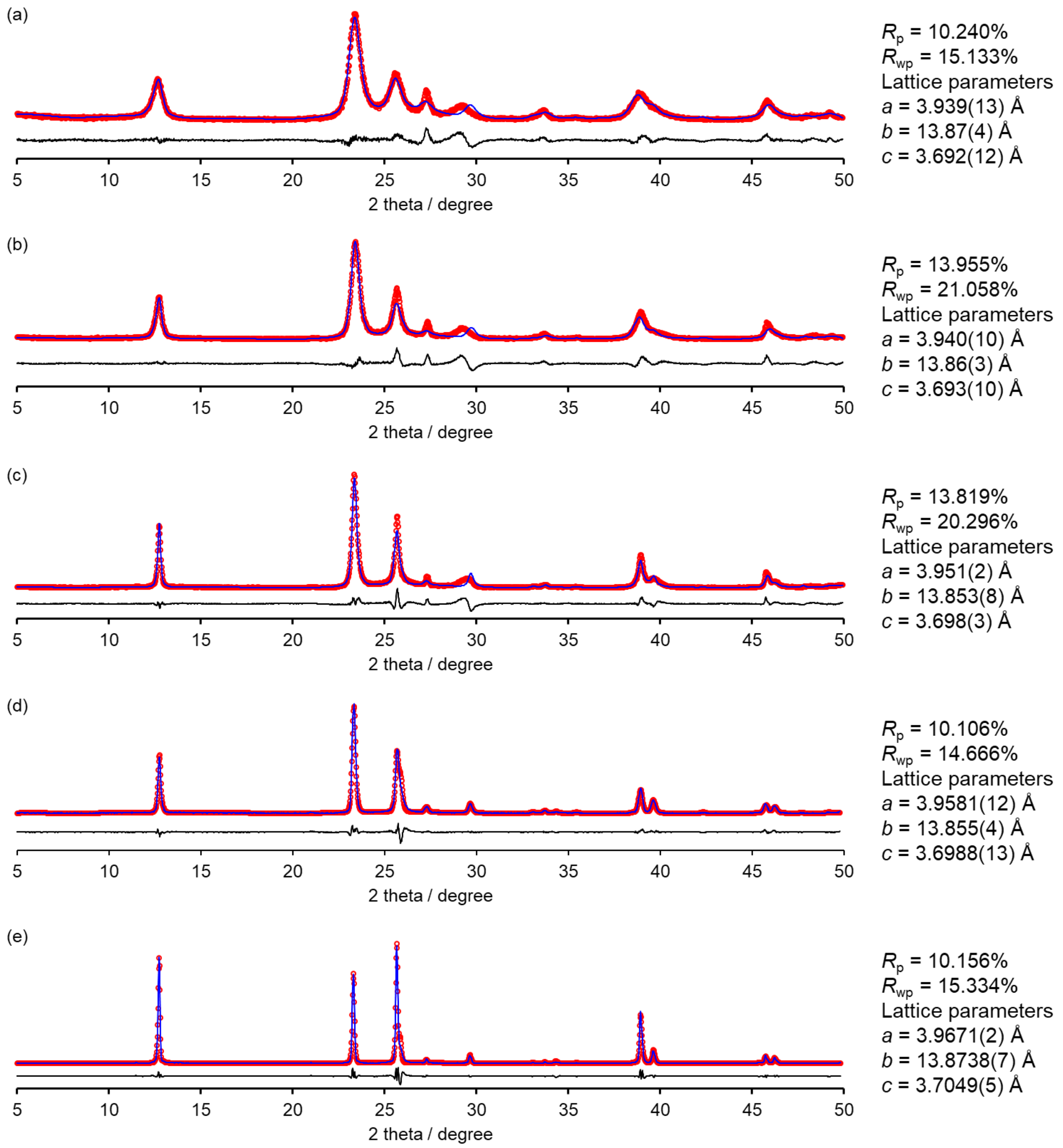


**Figure S17.** Le Bail refinements of the XRD patterns of (a) $\mathbf{1}_{300}$, (b) $\mathbf{1}_{350}$, (c) $\mathbf{1}_{400}$, (d) $\mathbf{1}_{500}$, and (e) $\mathbf{1}_{600}$ using EXPO2014.[S7] The overlying red circles and solid blue lines represent the calculated and observed intensities. The solid black lines represent the residual intensities. The fittings did not converge well because of the highly anisotropic nature of fibrous crystals $\mathbf{1}_n$ and the formation of nanorods.

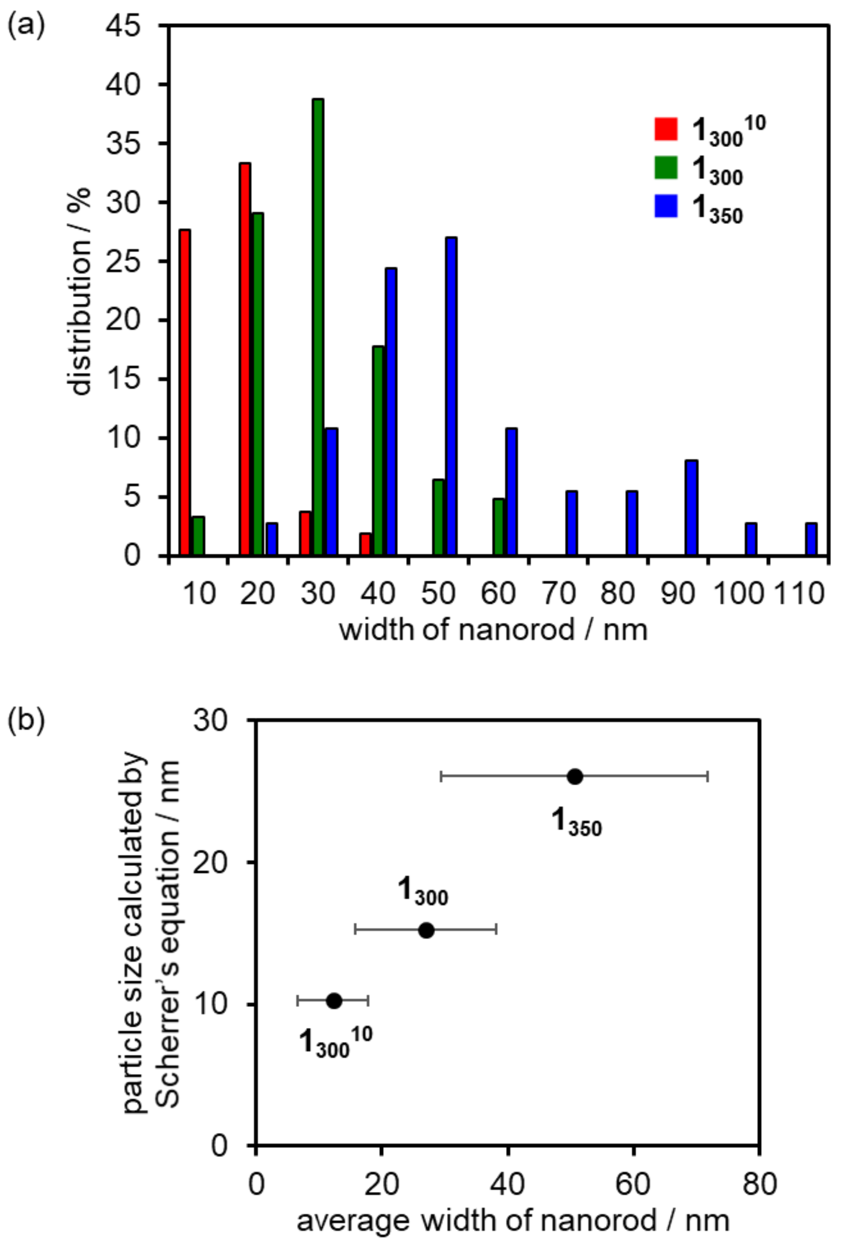


**Figure S18.** (a) Width distribution of nanorods in $\mathbf{1}_{300}{}^{10}$, $\mathbf{1}_{300}$, and $\mathbf{1}_{350}$ determined from the TEM images using 58, 62 and 37 nanorods, respectively. It should be noted that widths in densely aggregated nanorods were hardly measured because it is not clear to judge whether condensation reaction occurred between nanorods. Therefore, only tips of submicroribbons with clearly separated nanorods were utilized to determine widths. (b) Plot of average width of nanorods with standard deviation versus particle size calculated by Scherrer's equation using the FWHM of 020 reflections and K value of 1.0747 to compare observed and calculated values.

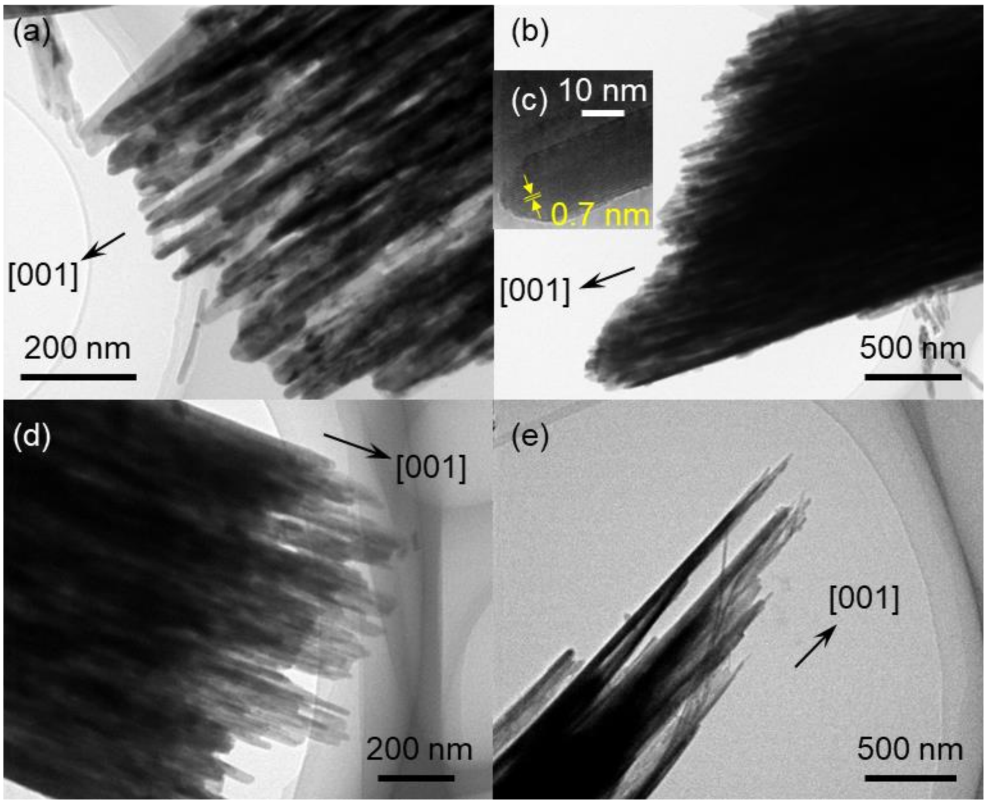


**Figure S19.** (a–c) TEM images of $\mathbf{1}_{300}$, where the image c is a nanorod of the bundle in the image b. (d,e) TEM images of $\mathbf{1}_{220}$.

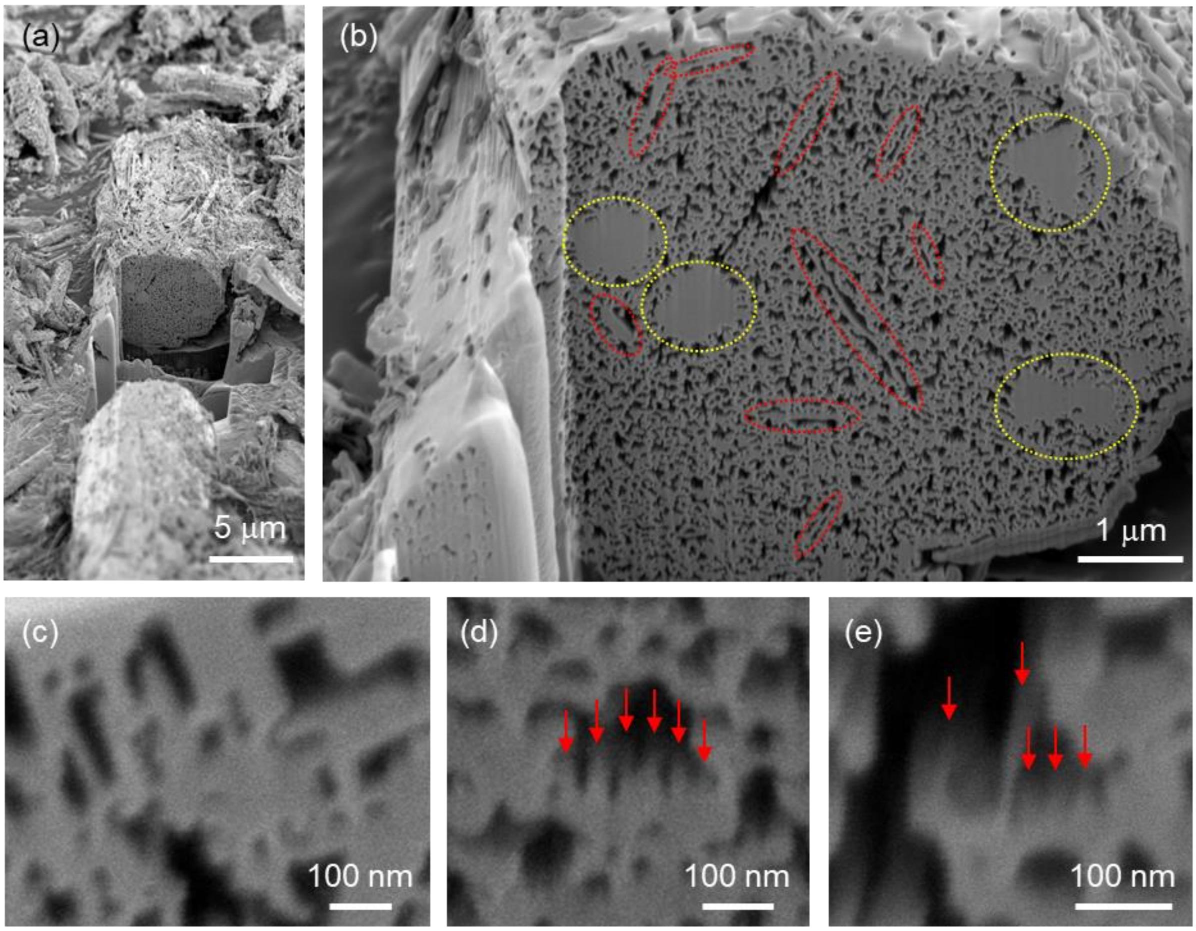


**Figure S20.** FIB-SEM images of **1**$_{300}$. (a) Whole image of a microfiber. (b) Cross section of the microfiber. Red and yellow ovals represent selected large plate-shaped and lump domains, respectively. (c–e) Enlarged images of the cross section b. Red arrows represent the tops of ridge-like structures.

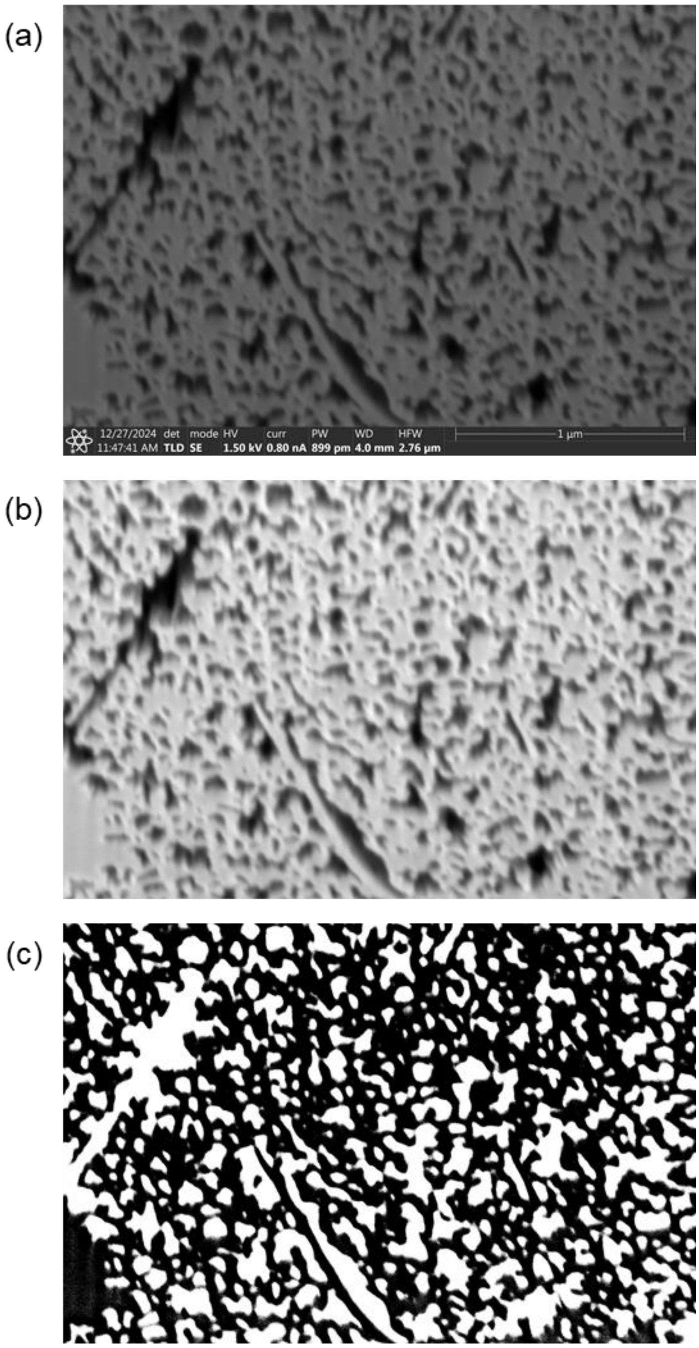


**Figure S21.** (a) Original, (b) image-processed, and (c) binarized FIB-SEM images of the cross section of $\mathbf{1}_{300}$. The ratio of white and black area in the image c (41:59) was determined by ImageJ version 1.54m.[S8]

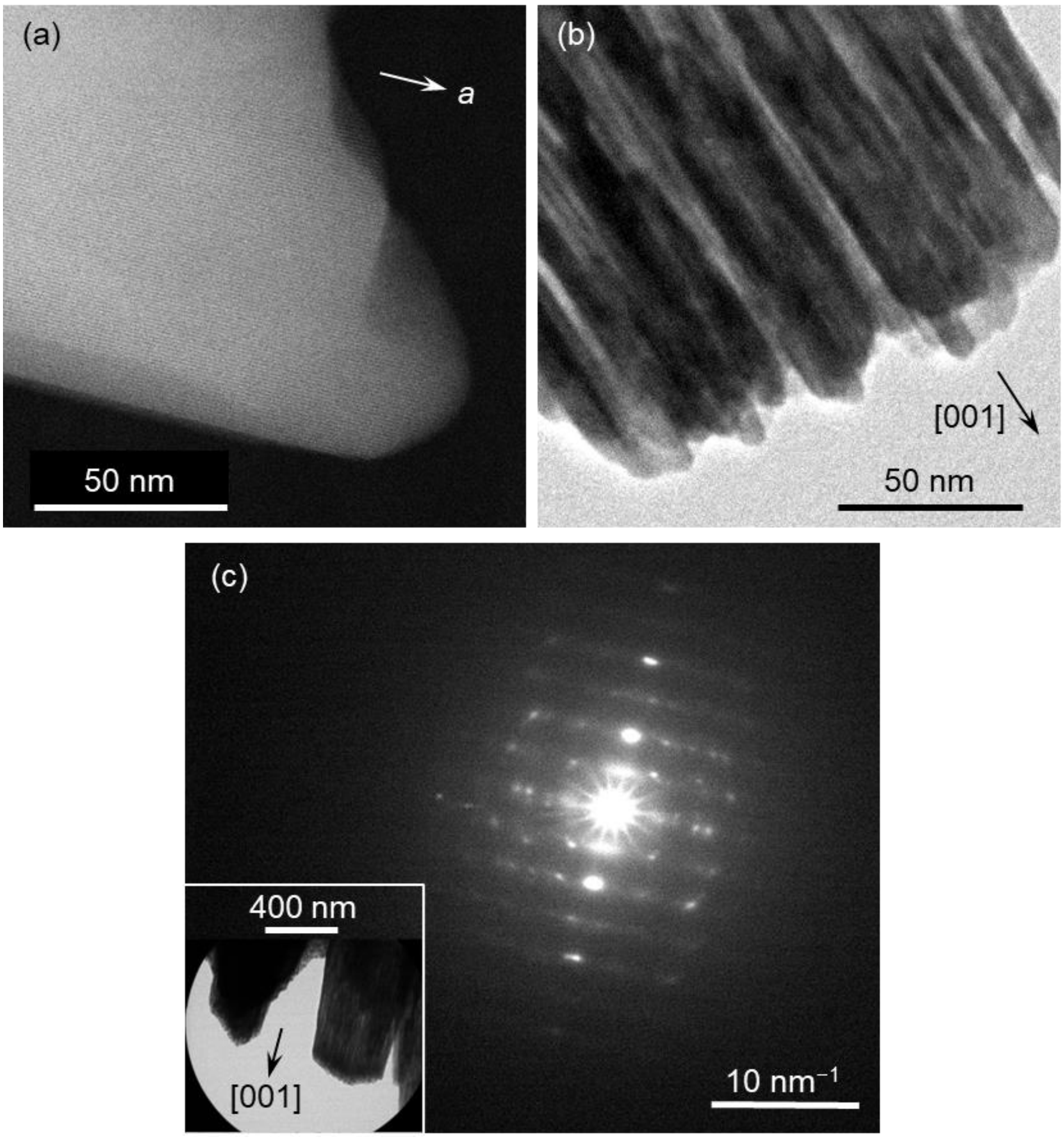


**Figure S22.** (a) STEM image of the tip of a microfiber **1**. (b) TEM image of the tip of a microfiber $\mathbf{1_{300}^{10}}$. (c) TEM image and corresponding SAED pattern of $\mathbf{1_{300}^{10}}$.

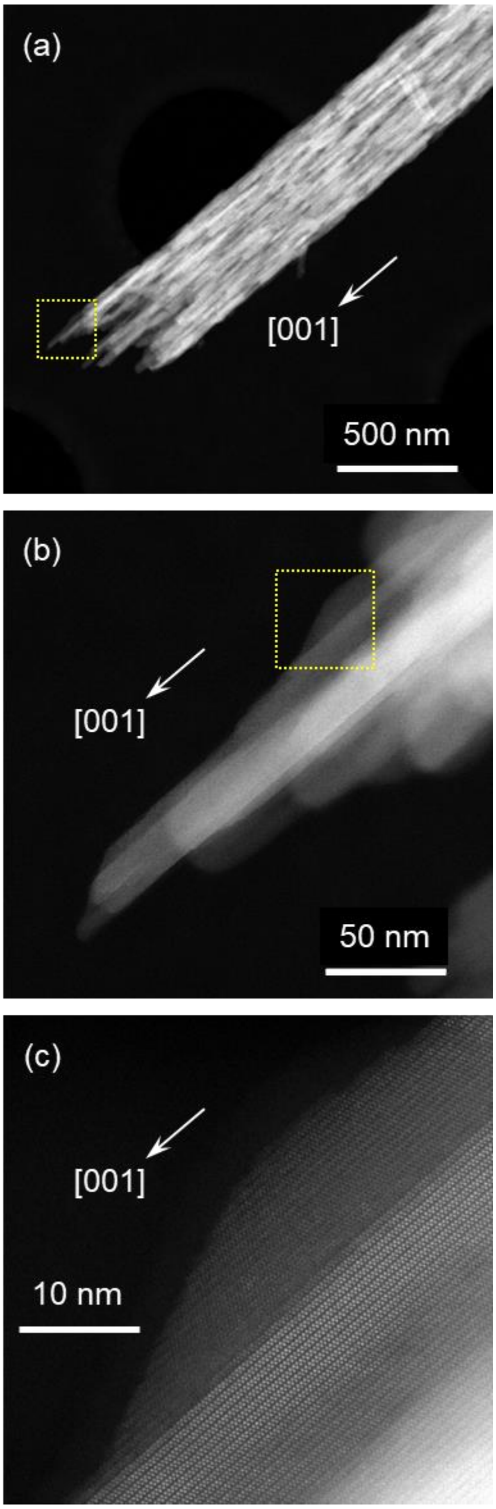


**Figure S23.** (a) STEM image of a submicroribbon $\mathbf{1}_{300}$. (b,c) Enlarged views of the images a and b, respectively. Image c is the same image of Figure 5g.

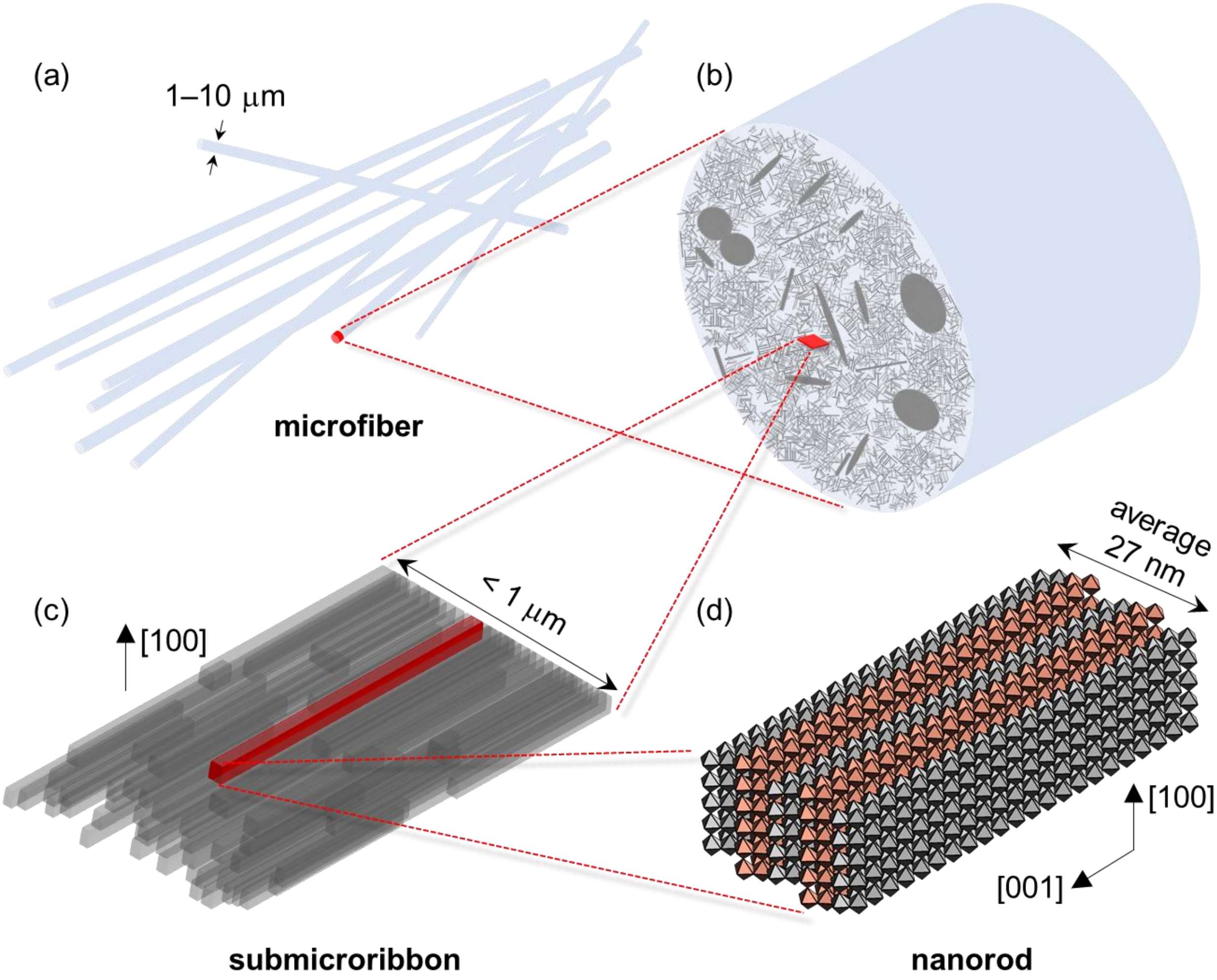


**Figure S24.** Schematic of hierarchical structures of $\mathbf{1}_{300}$: (a) microfibers, (b) the cross section of a microfiber, (c) submicroribbon, and (d) nanorod, where 2D sheets of α-$MoO_3$ are colored by grey and pale red alternately for clarity. Note that actual numbers of layers along the [010] direction would be around 40 layers for 27 nm.

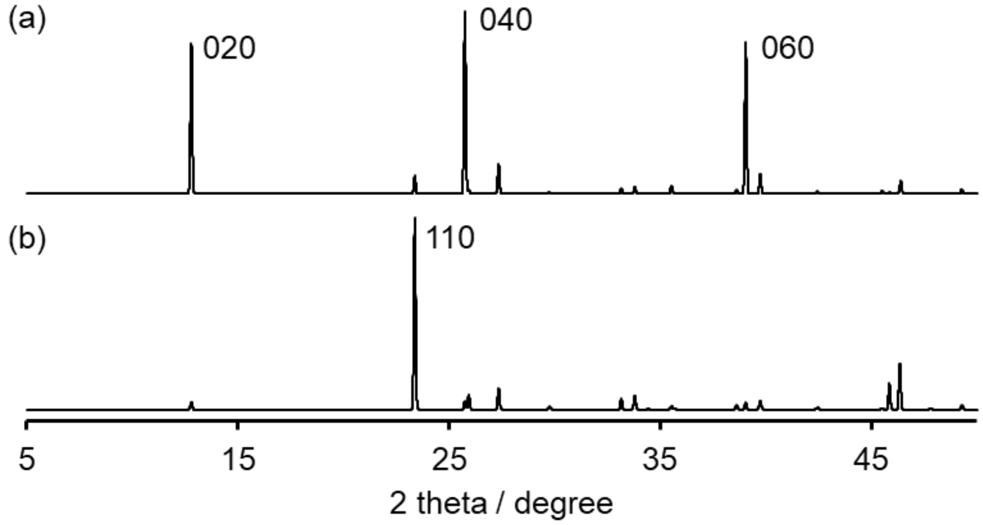


**Figure S25.** Simulated XRD patterns of α-$MoO_3$ by assuming plate-type crystal orientations along (a) the [010] direction and (b) the [100] direction with the alignment factors of 0.5. These patterns clearly indicated that the XRD pattern of nanorods $\mathbf{1_{300}}$ with the (100) plane as the broad face should show the strongest diffraction peak of 110 reflection. It should be noted that diffraction peaks from {100} plane cannot be observed due to the systematic absences.

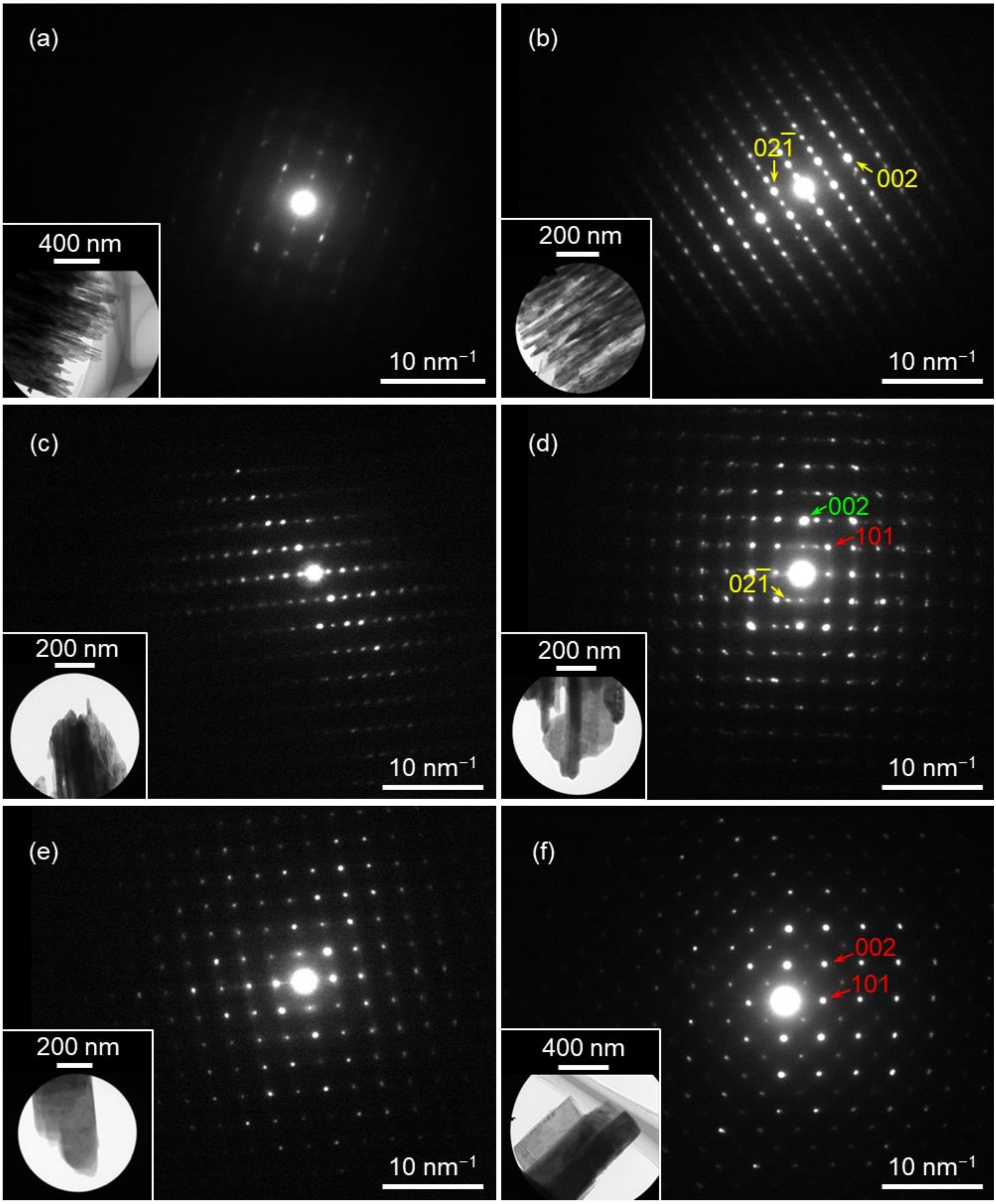


**Figure S26.** TEM images and corresponding SAED patterns of (a) $\mathbf{1_{220}}$, (b) $\mathbf{1_{300}}$, (c) $\mathbf{1_{350}}$, (d) $\mathbf{1_{400}}$, (e) $\mathbf{1_{500}}$, and (f) $\mathbf{1_{600}}$. Reflections in yellow, red, and green represent the reflections from the (100), (010), and both (100) and (010) planes, respectively.

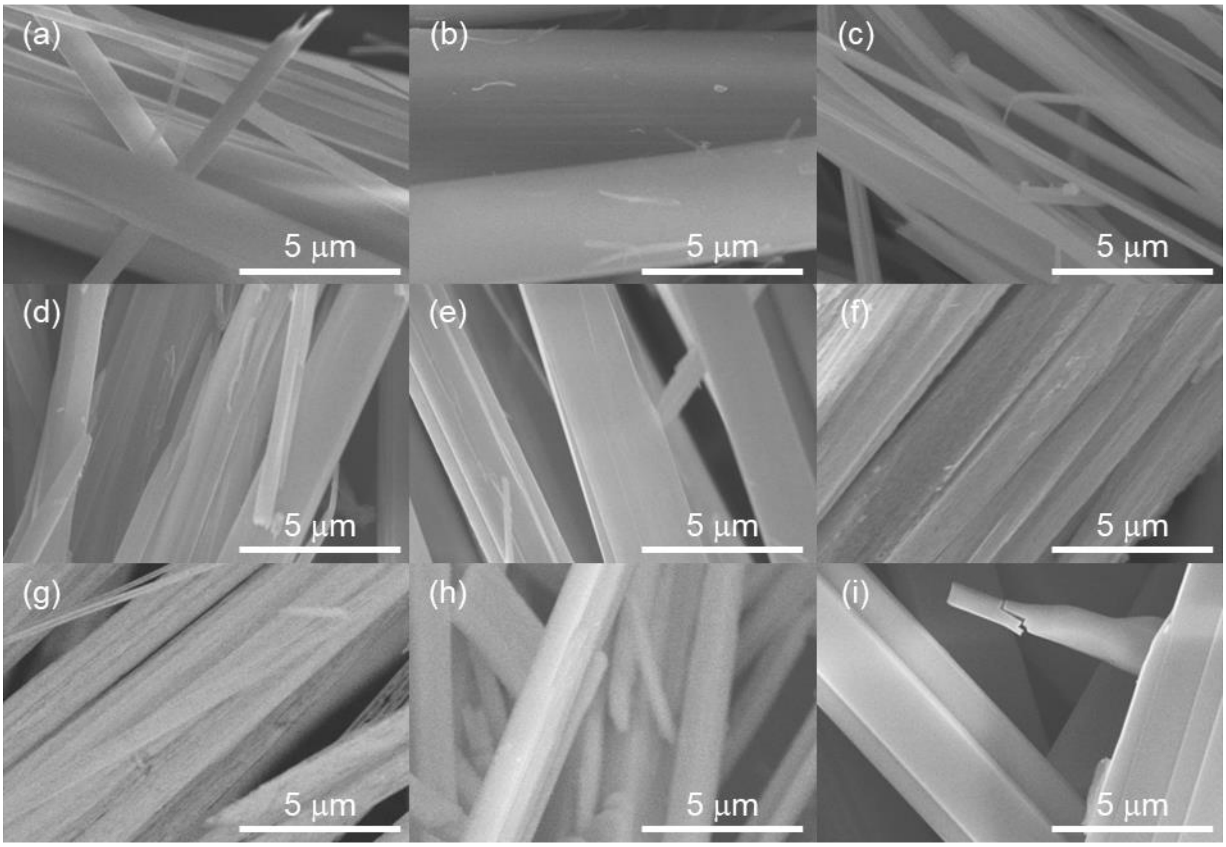


**Figure S27.** SEM images of (a) **1**, (b) $\mathbf{1}_{150}$, (c) $\mathbf{1}_{200}$, (d) $\mathbf{1}_{250}$, (e) $\mathbf{1}_{300}$, (f) $\mathbf{1}_{350}$, (g) $\mathbf{1}_{400}$, (h) $\mathbf{1}_{500}$, and (i) $\mathbf{1}_{600}$.

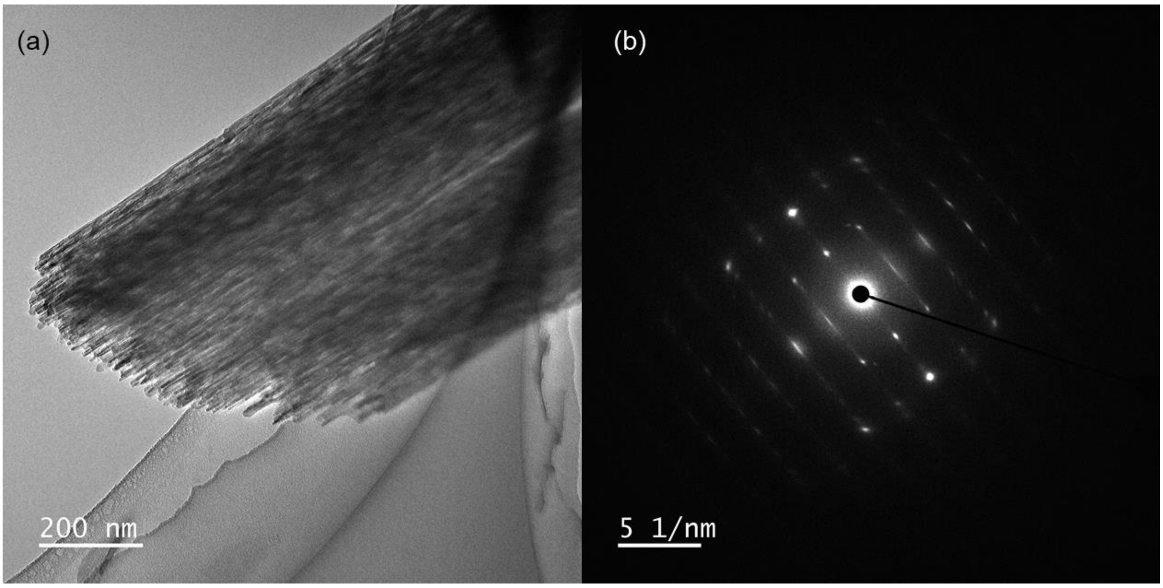


**Figure S28.** (a) TEM image and (b) corresponding SAED pattern of $\mathbf{1}_{\mathbf{300}}^{\mathbf{6}}$.

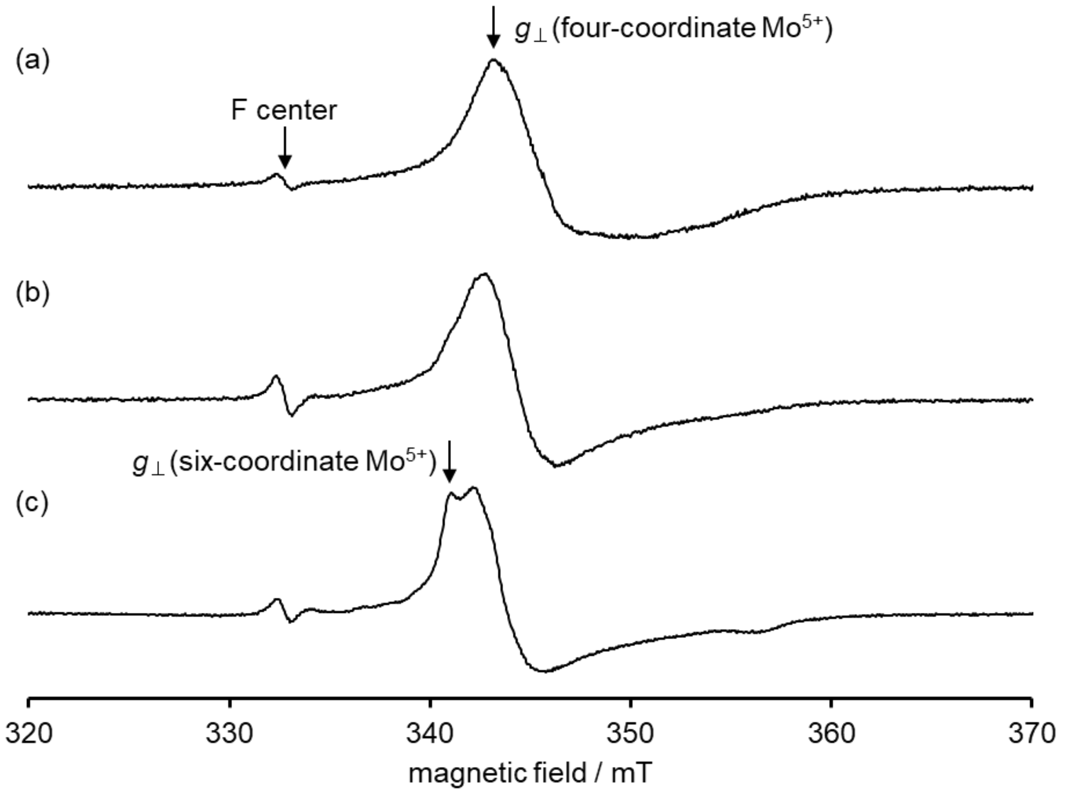


**Figure S29.** ESR spectra of (a) $\mathbf{1_{300}^{6}}$, (b) $\mathbf{1_{300}^{8}}$, and (c) $\mathbf{1_{300}^{10}}$ recorded at 77 K. It should be noted that signals assignable to five-coordinate $Mo^{5+}$ centers are not clearly observed at 77 K and observable at 300 K.[S9]

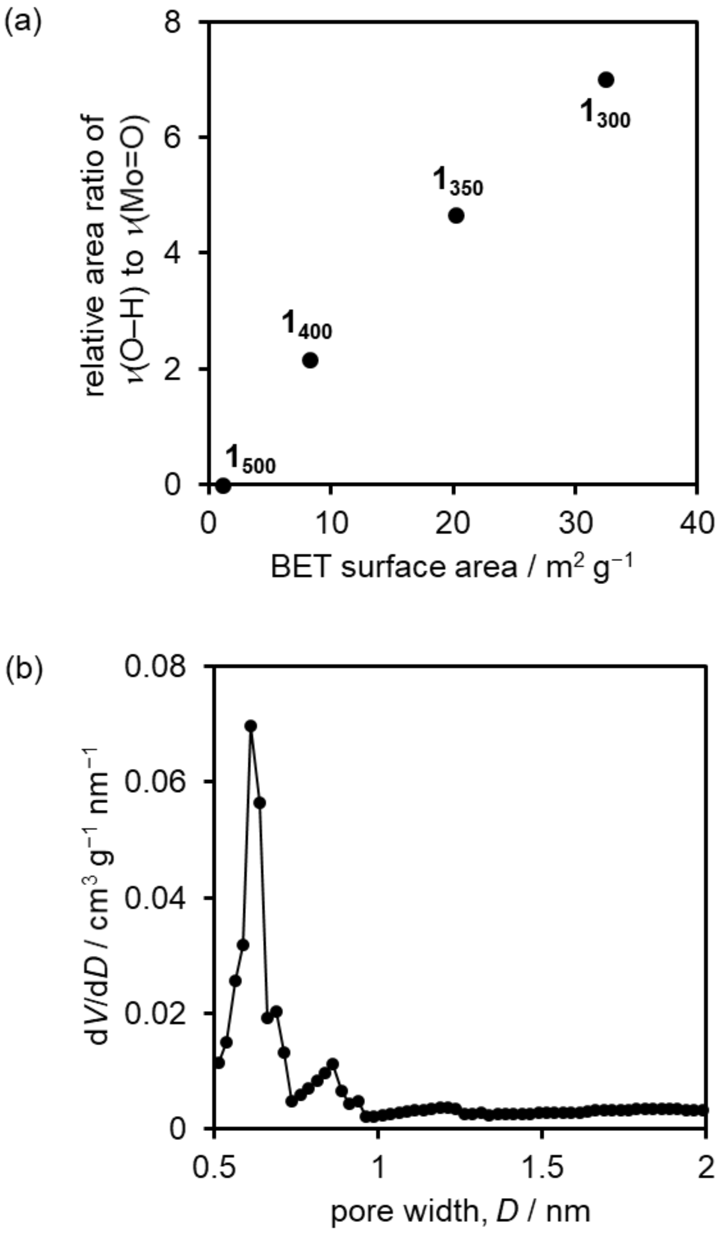


**Figure S30.** (a) Plot of relative area ratio of the peaks of $\nu$(O–H) to those of $\nu$(Mo=O) in the IR spectrum of $\mathbf{1}_{300}$. (b) HK plot of $\mathbf{1}_{300}$.

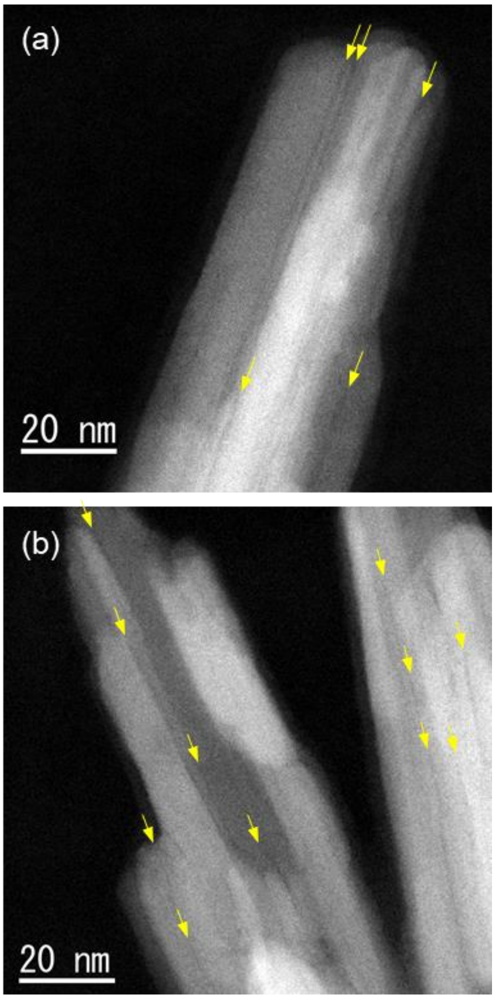


**Figure S31.** (a,b) STEM images of $\mathbf{1}_{300}$. Yellow arrows represent slit-shaped micropores.

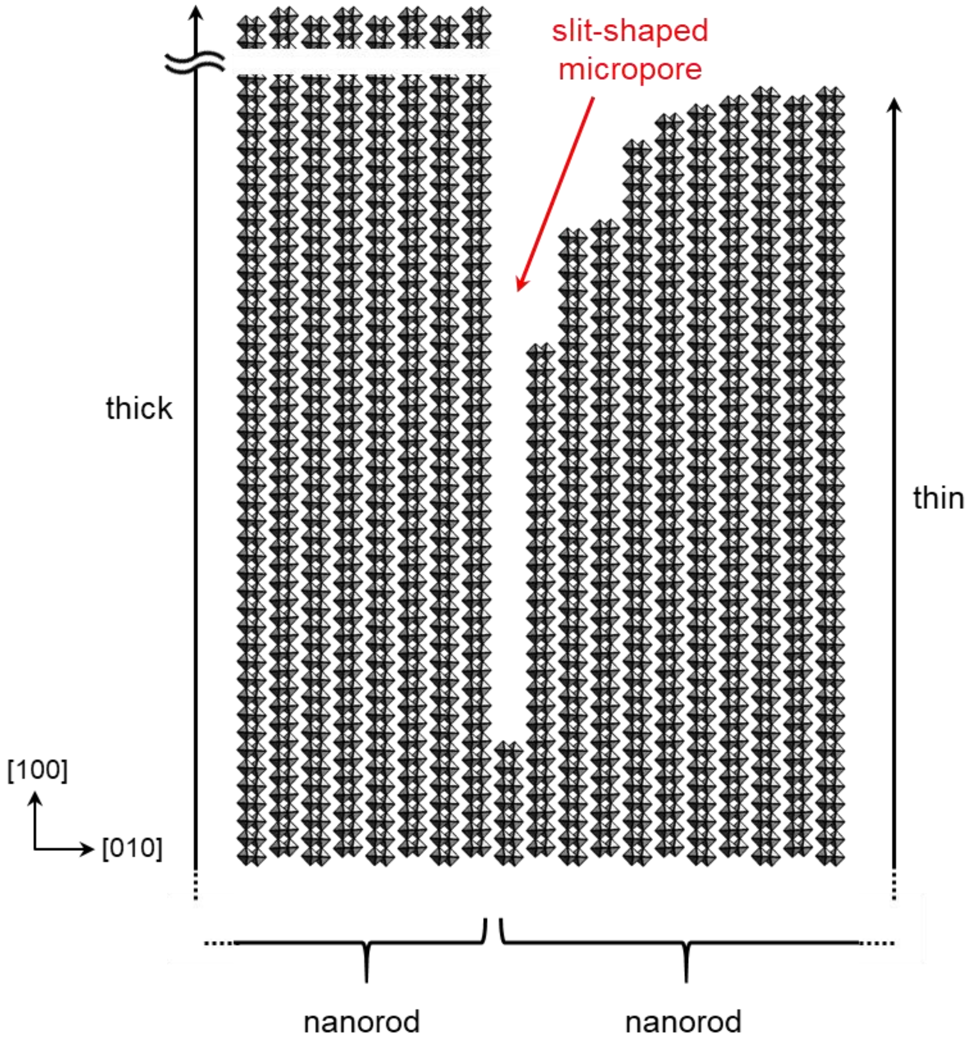


**Figure S32.** Expected cross section of the boundary of two nanorods in submicroribbon $\mathbf{1}_{300}$.

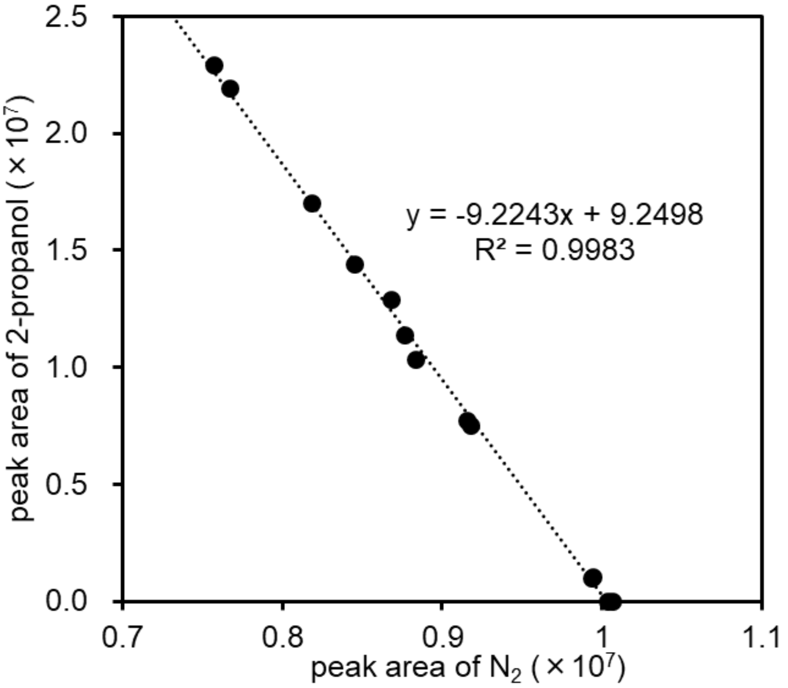


**Figure S33.** The standard calibration curve of 2-propanol. See the Experimental Section for the details.

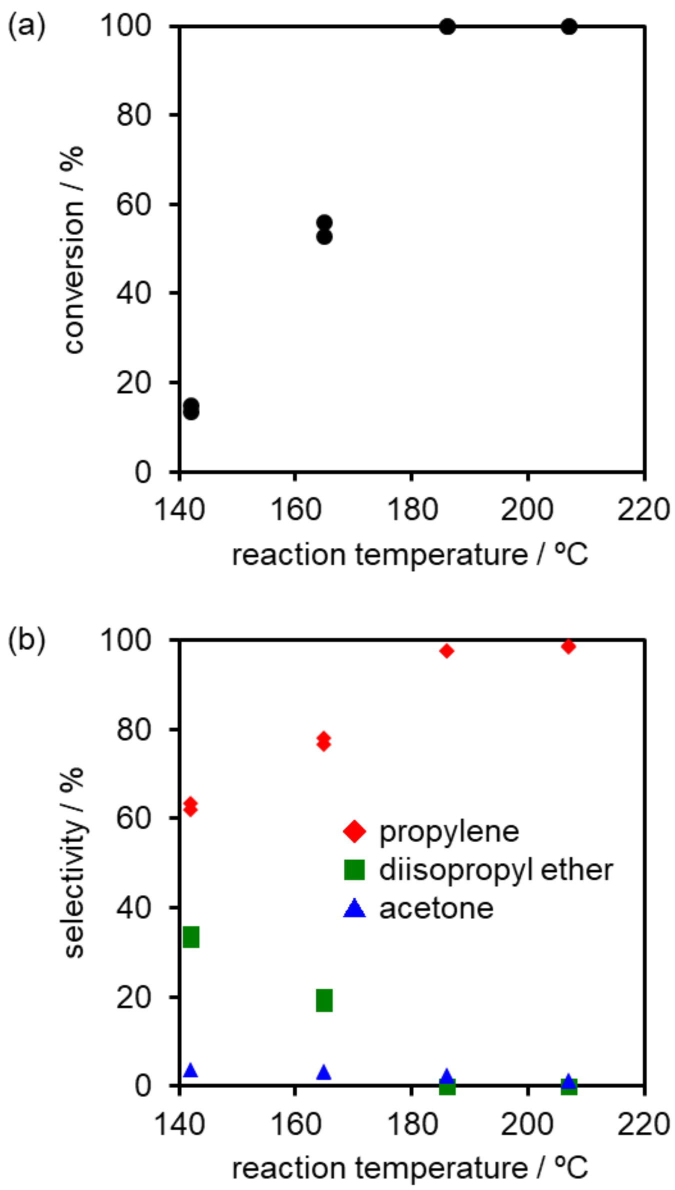


**Figure S34.** (a) Conversion and (b) selectivity in the catalytic dehydration reaction using $\mathbf{1}_{300}$.

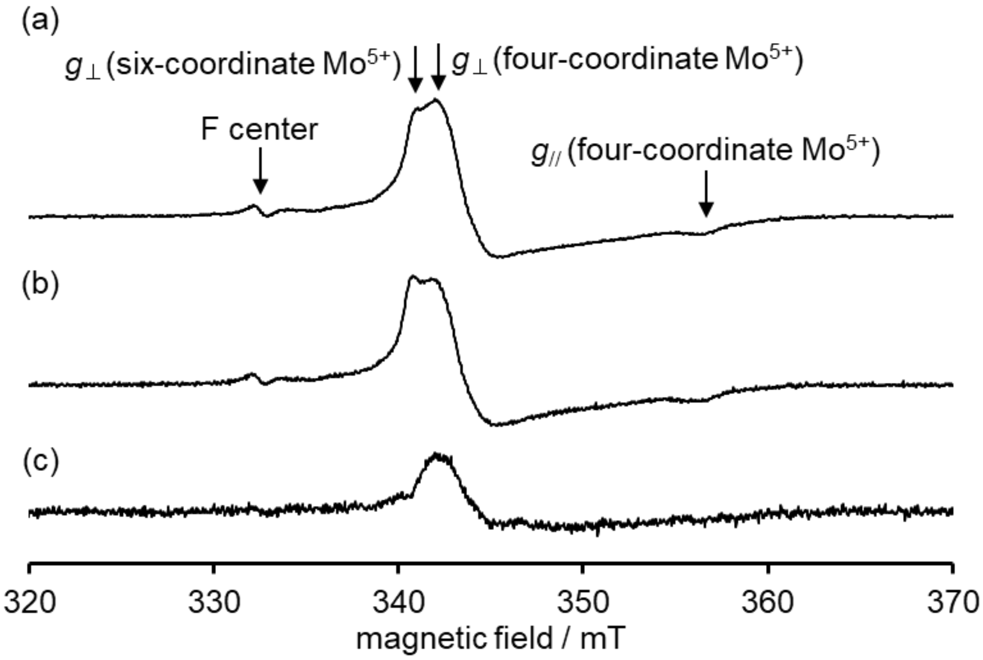


**Figure S35.** ESR spectra of $\mathbf{1}_{300}$ at 77 K after vacuum drying at 473 K for 10 min. (a) Before and (b) after addition of vaper of 2-propanol. (c) Differential spectrum obtained by subtracting the spectrum b from a. It should be noted that signals assignable to five-coordinate $Mo^{5+}$ centers are not clearly observed at 77 K and observable at 300 K.[S9]

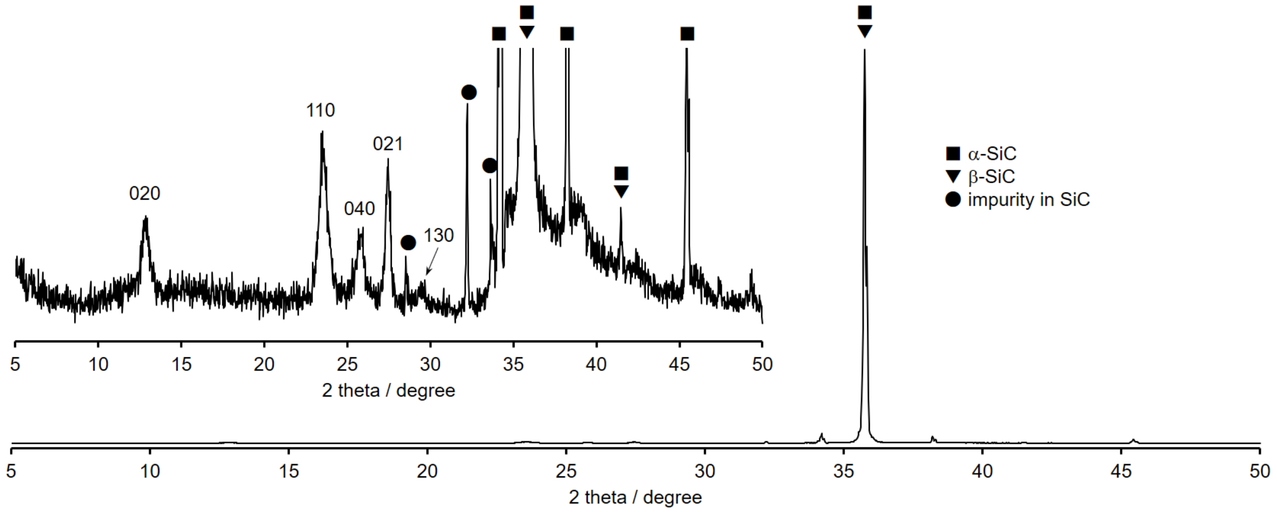


**Figure S36.** XRD pattern of the catalyst $\mathbf{1}_{300}$ after the continuous catalytic reaction for 24 h. Indexed peaks were attributed to $\alpha$-$MoO_3$.

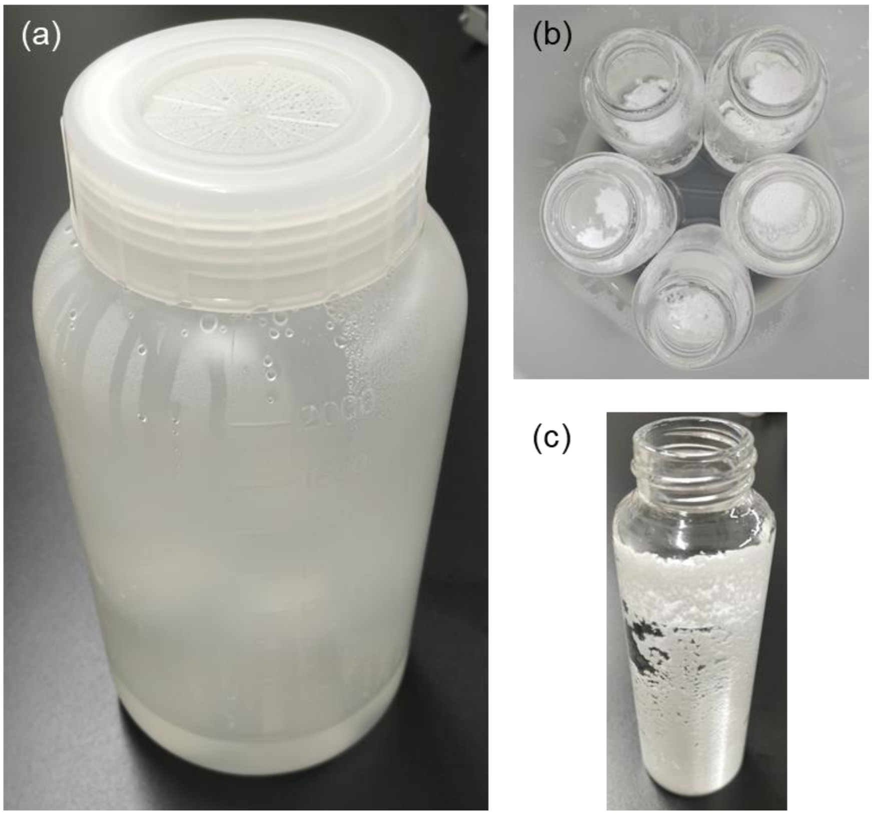


**Figure S37.** Bottles for crystallization of **1** used in this study. (a) DMF in a large plastic bottle (2 L). (b) Crystallization solutions in five glass bottles within a plastic bottle. (c) Fibrous crystals **1** formed in a glass bottle.

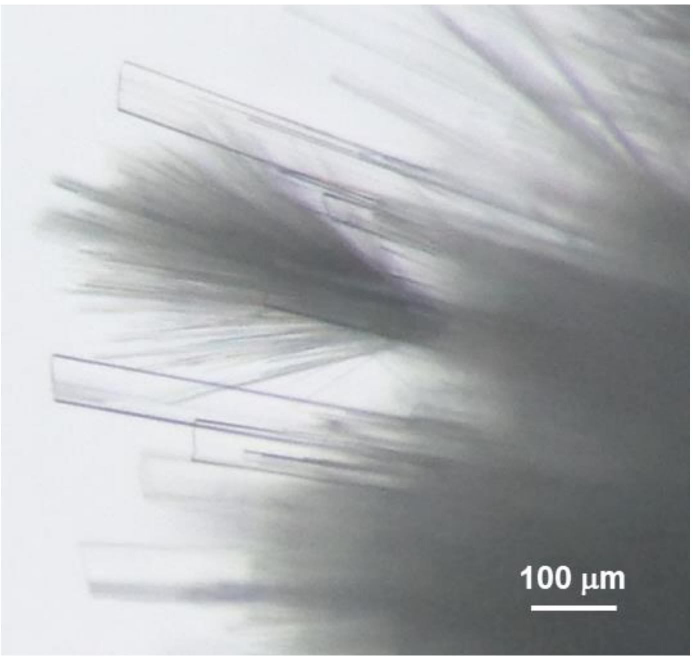


**Figure S38.** Optical microscopy image of single crystals **1** suitable for X-ray crystallographic analysis.

**References**


[S1] Bruker, *APEX3, SADABS, SAINT* **2016**.

[S2] L. J. Farrugia, *J. Appl. Cryst.* **1999**, *32*, 837.

[S3] G. M. Sheldrick, *Acta Cryst.* **2015**, *A71*, 3.

[S4] G. M. Sheldrick, *Acta Cryst.* **2015**, *C71*, 3.

[S5] N. E. Brese, M. O'Keeffe, *Acta Cryst.* **1991**, *B47*, 192.

[S6] M. K. Trivedi, A. Branton, D. Trivedi, G. Nayak, G. Saikia, S. Jana, *Am. J. Appl. Chem.* **2015**, *3*, 188.

[S7] A. Altomare, C. Cuocci, C. Giacovazzo, A. Moliterni, R. Rizzi, N. Corriero, A. Falcicchio, *J. Appl. Cryst.* **2013**, *46*, 1231.

[S8] Rasband, W.S., ImageJ, U. S. National Institutes of Health, Bethesda, Maryland, USA, https://imagej.net/ij/, 1997-2018.

[S9] C. Louis, M. Che, *J. Phys. Chem.* **1987**, *91*, 2875.
